# The role of muscle degeneration and spinal balance in the pathophysiology of lumbar spinal stenosis: Study protocol of a translational approach combining in vivo biomechanical experiments with clinical and radiological parameters

**DOI:** 10.1371/journal.pone.0293435

**Published:** 2023-10-27

**Authors:** David Koch, Corina Nüesch, Dominika Ignasiak, Soheila Aghlmandi, Alice Caimi, Guido Perrot, Friederike Prüfer, Dorothee Harder, Francesco Santini, Stefan Schären, Stephen Ferguson, Annegret Mündermann, Cordula Netzer

**Affiliations:** 1 Department of Spine Surgery, University Hospital Basel, Basel, Switzerland; 2 Department of Biomedical Engineering, University of Basel, Basel, Switzerland; 3 Department of Orthopaedics and Traumatology, University Hospital Basel, Basel, Switzerland; 4 Institute for Biomechanics, ETH Zurich, Zurich, Switzerland; 5 Division of Clinical Epidemiology, University of Basel and University Hospital Basel, Basel, Switzerland; 6 Department of Physiotherapy, University Hospital Basel, Basel, Switzerland; 7 Department of Pediatric Radiology, University Children’s Hospital Basel, Basel, Switzerland; 8 Department of Radiology, University Hospital Basel, Basel, Switzerland; UNITED KINGDOM

## Abstract

**Objective:**

To describe a study protocol for investigating the functional association between posture, spinal balance, ambulatory biomechanics, paraspinal muscle fatigue, paraspinal muscle quality and symptoms in patients with symptomatic lumbar spinal stenosis (sLSS) before and 1-year after elective surgical intervention.

**Design:**

Single-centre prospective, experimental, multimodal (clinical, biomechanical, radiological) study with three instances of data collection: baseline (study visit 1), 6-month follow-up (remote) and 1-year follow-up (study visit 2). Both study visits include an *in vivo* experiment aiming to elicit paraspinal muscle fatigue for postural assessment in a non-fatigued and fatigued state.

**Experimental protocol:**

At baseline and 1-year follow-up, 122 patients with sLSS will be assessed clinically, perform the back-performance scale assessment and complete several patient-reported outcome measure (PROMs) questionnaires regarding overall health, disease-related symptoms and kinesiophobia. Posture and biomechanical parameters (joint kinematics, kinetics, surface electromyography, back curvature) will be recorded using an optoelectronic system and retroreflective markers during different tasks including overground walking and movement assessments before and after a modified Biering-Sørensen test, used to elicit paraspinal muscle fatigue. Measurements of muscle size and quality and the severity of spinal stenosis will be obtained using magnetic resonance imaging (MRI) and sagittal postural alignment data from EOS radiographies. After each study visit, physical activity level will be assessed during 9 days using a wrist-worn activity monitor. In addition, physical activity level and PROMs will be assessed remotely at 6-month follow-up.

**Conclusion:**

The multimodal set of data obtained using the study protocol described in this paper will help to expand our current knowledge on the pathophysiology, biomechanics, and treatment outcome of degenerative sLSS. The results of this study may contribute to defining and/or altering patient treatment norms, surgery indication criteria and post-surgery rehabilitation schedules.

**Trial registration:**

The protocol was approved by the regional ethics committee and has been registered at clinicaltrials.gov (NCT05523388).

## Introduction

Spinal claudication due to lumbar spinal stenosis (LSS) is a clinical syndrome of the ageing human spine. LSS is characterized by age-related degeneration of the lumbar discs [1], facet joints (FJs) [2], and hypertrophy of the ligamentum flavum [3,4], leading to pain [5], limited function [6], and compromised quality of life [7]. Symptomatic LSS (sLSS) is an often severely disabling condition [8], the most common reason for spinal surgery in patients over 65 years of age [9,10], and represents a major financial burden to the health care system and society [11]. SLSS has been associated with disability [12], atrophy and fatty infiltration of paraspinal muscles [13], decreased physical activity [14], walking capacity [12,15], altered gait patterns and postural balance [15–19], as well as changes in sagittal balance of the spine [20]. However, the natural course and the pathophysiological processes of sLSS are not fully understood.

The overall function of the spine is determined by the interrelationship between the pelvis, the sacrum and the local curvatures of the lumbar, thoracic and cervical spine [21]. In a healthy spine, the local curvatures result in a state of physiological alignment that requires minimal muscle activity to maintain upright stance [22]. The resulting state is referred to as global spinal balance [23]. In this state, global and local loads on the spine have minimal effects on the diameter of the spinal canal during static posture and dynamic motion. However, global spinal balance in patients with sLSS may be compromised due to the narrowing in the spinal canal [20]. To evade typical pain symptoms caused by the narrowing, it is believed that patients adopt a compensated posture, typically decreasing the lumbar lordosis in an attempt to open the spinal canal.

Clinical observations have shown that the presence and intensity of symptoms depend on the body’s posture (e.g., exacerbated when standing, relieved when sitting or lying) or activity (e.g., exacerbated when walking, relieved when bending forward) [24]. These observations suggest an influence on function and the importance of a physiological alignment of the spine. Hence, understanding the relationship between spinal kinematics and functional disability is one of the key factors to understand the natural course of this disease. Although these observations clearly demonstrate that spinal motion plays a critical role in clinical presentation of LSS, to date dynamic *in vivo* spinal kinematics during activities of daily living in patients with sLSS are largely unknown. Based on clinical observations and our previous work [25–29], the following research questions were raised and will be answered in this project:

- Are patient reported outcome measures (PROMs) related to severity of stenosis, spinal alignment, segmental instability, muscle degeneration, dynamic compensation, muscle fatigue and spinal posture and motion?
- Do radiologically described changes in muscle quality correlate with fatigue exercise duration and paraspinal muscle fatigue during the modified Biering-Sørensen test?
- Does paraspinal muscle fatigue affect posture and gait biomechanics in patients with sLSS?
- Do PROMs, spinal alignment, muscle degeneration, dynamic compensation, muscle fatigue, physical activity level, and spinal posture and motion improve after spinal stenosis surgery?
- Do pre- to postoperative changes in PROMs correlate with pre- to postoperative changes in spinal alignment, muscle degeneration, dynamic compensation, muscle fatigue, physical activity level, and spinal posture and motion?

This study primarily investigates the association between postural and ambulatory biomechanics and symptoms in patients with sLSS. In the *in vivo* experiment, we will use motion analysis and electromyography (EMG) to study the functional association between paraspinal muscle fatigue, posture, and gait. Moreover, we seek to explore the effect of sLSS on paraspinal muscle quality using magnetic resonance imaging (MRI) and the effect of sLSS on sagittal spinal balance using EOS radiography. Finally, the effect of routine surgical intervention on parameters collected pre-and postoperatively will be evaluated. We will address the following specific aims:

### Specific Aim 1

*Establish the relationship between PROMs, spinal alignment and imbalance, dynamic compensation, severity of stenosis, segmental instability, muscle degeneration and fatigue, and biomechanical parameters in patients with sLSS*.

Hypothesis 1.1: PROMs correlate with the extent of dynamic compensation (difference between static and dynamic spinal alignment) in patients with sLSS.
Hypothesis 1.2: PROMs correlate more strongly with quantitative functional parameters including muscle fatigue and biomechanical parameters or lumbopelvic range of motion during forward trunk bending and during walking than with pathomorphological parameters including severity of stenosis, segmental instability, and muscle degeneration.
Hypothesis 1.3: Greater muscle fatigue in patients with sLSS is associated with a greater dynamic compensation.

A relation between PROMs, spinal alignment and spinal imbalance, muscle degeneration, dynamic compensation, muscle fatigue, severity of stenosis, and biomechanical parameters is the basic implicit assumption about the clinical relevance of these factors. This will be checked by correlating PROMs with clinical parameters including spinal imbalance, severity of stenosis, and muscle degeneration and with quantitative functional parameters including dynamic compensation, muscle fatigue and biomechanical parameters and extent of forward trunk bending and lumbopelvic range of motion during walking. In addition, the role of segmental instability and dynamic compensation will be depicted resulting potentially in a further subcategorization of patient groups. While the EOS images will allow the study of the anatomical spinopelvic balance during stance, the motion capture technique will allow the assessment of both static posture during stance and dynamic posture and spinal balance during gait. The difference between the static and the dynamic spinal balance may reveal potential compensation effects during gait, called dynamic compensation. The compensation effects can be further corroborated by establishing a positive association between PROMs, muscle fatigue and degeneration, spinal alignment and extent of forward trunk bending and lumbopelvic range of motion during walking when adjusted for segmental instability. The magnitude of biological variation can give a hint to the uniformity of compensation effects across patients also considering potential spinal alignment because of degenerative changes. These analyses may also imply a reassessment of the results of Hypothesis 1.2, which ignores potential compensation effects.

### Specific Aim 2

*Assess PROMs, severity of stenosis, spinal alignment and imbalance, muscle degeneration, dynamic compensation, muscle fatigue, physical activity level and biomechanical parameters in patients with sLSS 12 months after spinal stenosis surgery*.

Hypothesis 2.1: At follow-up, patients with sLSS will have improved PROMs, less static and dynamic spinal imbalance and muscle fatigue, and less forward trunk bending and larger lumbopelvic range of motion during walking than before spinal stenosis surgery.
Hypothesis 2.2: Pre- to postoperative changes in PROMs correlate with pre- to postoperative changes in static and dynamic spinal imbalance, muscle fatigue and extent of forward trunk bending and lumbopelvic range of motion during walking in patients undergoing spinal stenosis surgery.

A relation between postoperative changes in PROMs and changes in spinal alignment, muscle fatigue and degeneration and biomechanical parameters would prove the assumption that surgery affects parameters that determine patient reported outcome. This is checked by correlating changes in PROMs with changes in spinal alignment, muscle fatigue and biomechanical parameters.

## Materials and methods

### Study design

This study is designed as a single-centre, longitudinal observational study. For each patient, multimodal data including clinical, functional, radiological, and biomechanical data will be collected before and after routine surgical intervention.

### Participants

We plan to enrol 122 patients with sLSS (61 per year) in this study. Patients will be recruited at the Department of Spine Surgery of the University Hospital Basel. Recruitment and data collection commenced on the 15^th^ of August 2022 and will continue until the recruitment target is met. Baseline data collection is expected to be completed in August 2024, while follow-up measurements are expected to be completed by the end of 2025.

The surgical procedure will not be influenced by the study and is not subject of investigation. All patients will receive–depending on clinical and imaging diagnosis–lumbar decompression alone or decompression in combination with fusion. Fusion will be achieved either by posterolateral fusion with a pedicle screw-rod system with autologous bone apposition only or by additional implantation of transforaminal lumbar interbody fusion with a cage filled with autologous bone implanted from posterolateral into the intervertebral space. Decompression will be performed either as open surgery or microsurgically, and both are performed through a midline approach. Open surgical decompression will be performed through an interspinous approach with interlaminar flavectomy, while the microsurgical technique involves unilateral or bilateral fenestration and flavectomy.

### Inclusion and exclusion criteria

Inclusion and exclusion criteria are listed in Table 1.

**Table 1:** Inclusion and exclusion criteria applied in this study.

Inclusion criteria	Exclusion criteria
Age > 30 years BMI < 35 kg/m^2^ Diagnosed sLSS Clinical symptoms for at least 6 months Intermittent neurogenic claudication with limitations of their walking ability due to symptoms in the lower back and/or in one or both legs Unsuccessful conservative treatment Confirmation of the LSS through MRI Scheduled for surgery	Inability to provide informed consent Previous spine surgery Use of walking aids Other neurologic disorders affecting gait MRI incompatibility Pregnancy

BMI–Body mass index; sLSS–symptomatic lumbar spinal stenosis; MRI–magnetic resonance imaging.

### Ethical considerations

The experimental protocol was approved (9th August 2022) by the regional ethics board (Ethics Committee Northwest and Central Switzerland, EKNZ 2022–01170) and registered at clinicaltrials.gov (NCT05523388). Study participation will be voluntary and written informed consent will be obtained from all participants prior to participation. Assessing the proposed study parameters in addition to standard clinical measures will provide additional insight into the functional limitations and the clinical course of the sLSS in our patients. This study involves a considerable time commitment for participants. However, the results of the study assessments will inform ongoing treatment planning, so this time investment may be outweighed by the benefits not only to future patients but also to study participants.

## Experimental protocol

### Procedures

**Fig 1:**
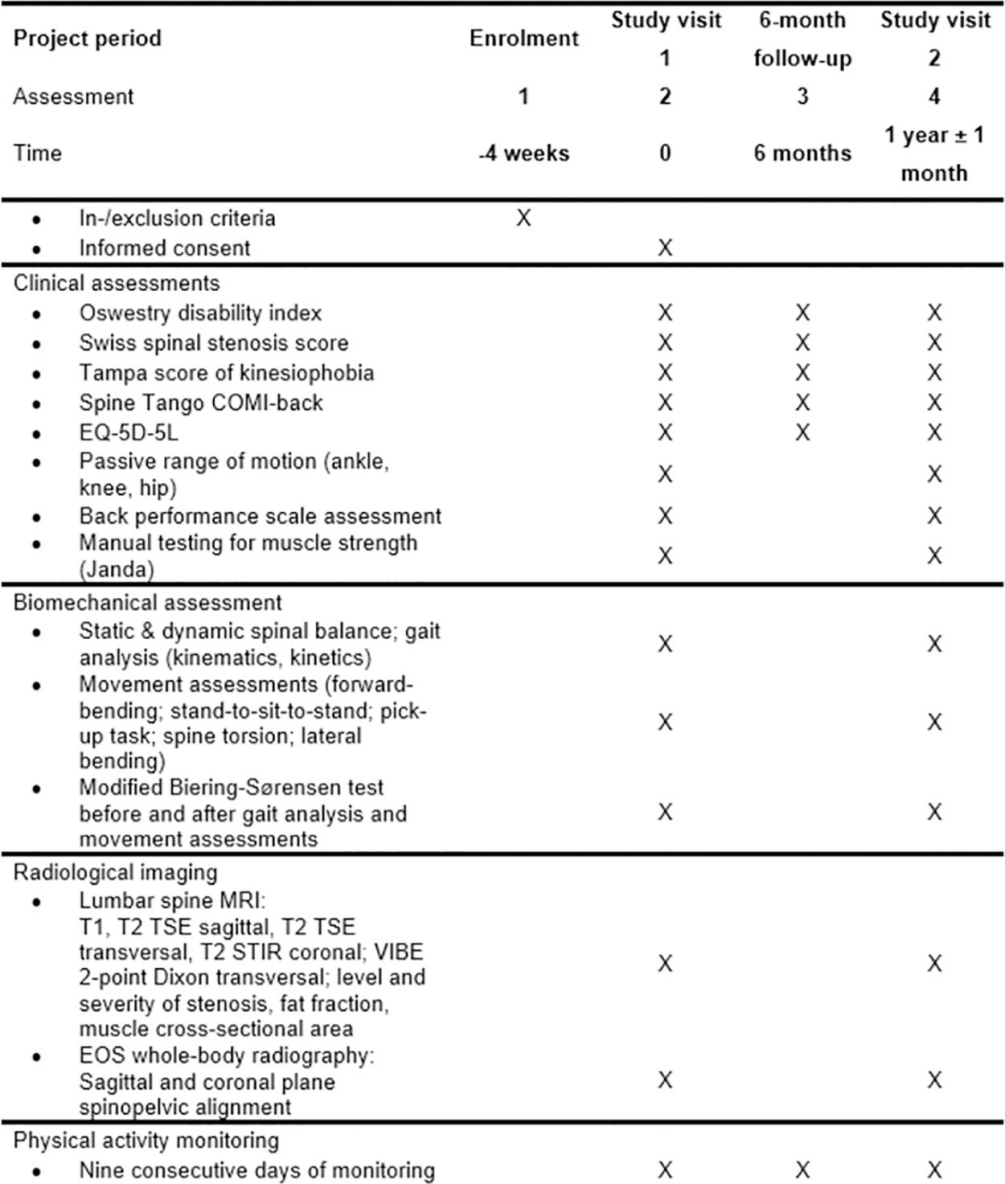
Schedule of enrolment and assessments.

**Fig 2:**
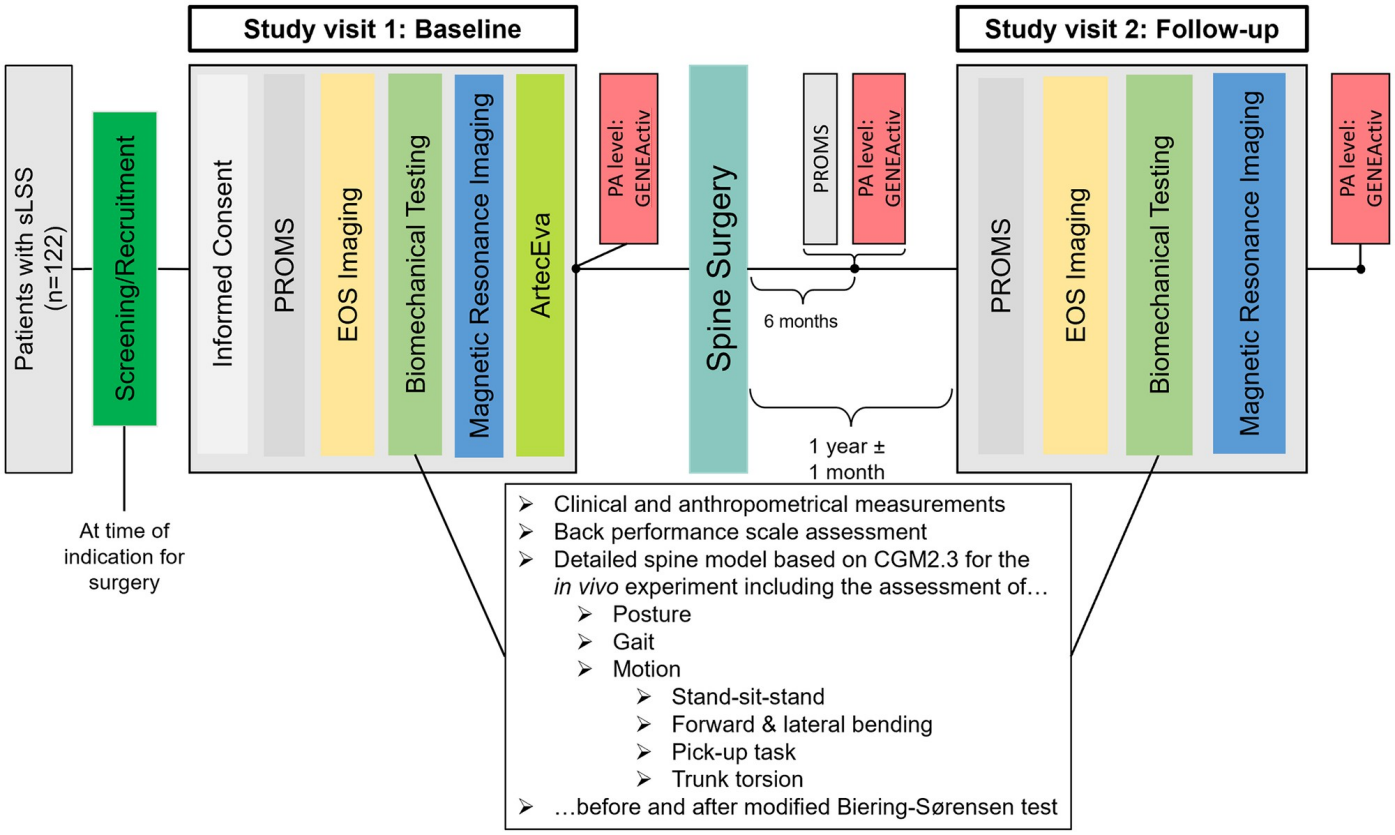
Study flow chart.

Data will be collected at three instances: at baseline (on-site study visit 1), at 6-month follow-up (remote data collection), and at 1-year follow-up (on-site study visit 2) (Fig 1). The same procedures will be performed during both on-site study visits (Fig 2). During both study visits, a comprehensive set of clinical, functional, biomechanical and radiological data will be collected. At 6-month follow-up, only PROMs and physical activity data will be collected.

At the beginning of study visit 1, patients will sign the informed consent form. Thereafter, participants will be asked to complete several PROMs questionnaires, including the Oswestry Disability Index (ODI) [30,31], EQ-5D-5L [32], Swiss Spinal Stenosis Questionnaire [33], Tampa scale of Kinesiophobia [34–36] and Spine Tango COMI [37].

In a next step, radiological data (MRI and EOS) will be collected. MRI of the lumbar and abdomen regions will be performed with patients lying supine with extended legs. Upright standing biplanar (sagittal and frontal) EOS images of the full body including the entire spine, pelvis, and lower extremity will be acquired. Ahead of the radiography, 15 radiopaque reference markers are placed on anatomical landmarks. For the duration of the radiography, patients will be asked to assume a specific position, which entails the elevation of the elbows to around 90°. Patients are asked to clench their fists and hold their hands to the sides of their cheeks. The position described ensures that the upper limbs do not obscure the spine during the radiography. This posture, called “EOS posture” will be referenced using a custom-made pose frame. After the radiography, the 3D back surface will be scanned using ArtecEva with the radiopaque reference markers still attached. For this purpose, patients will be brought back into the EOS position using the pose frame. After the scan, the reference markers will be removed and the patients prepared for the functional biomechanical analysis.

**Fig 3:**
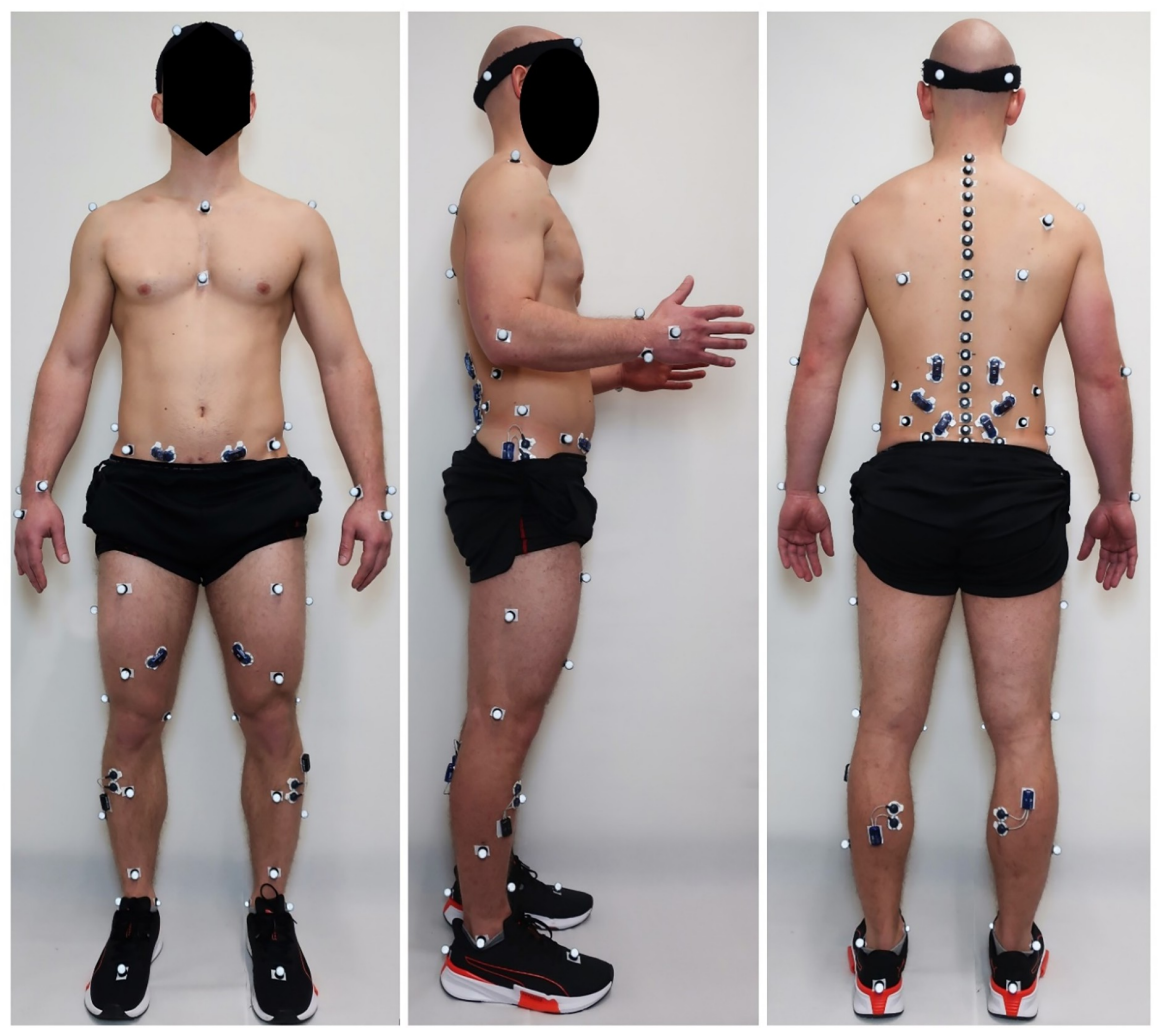
Placement of retroreflective marker and electromyography electrodes for the biomechanical assessment. Anterior (left), lateral (middle) and posterior (right) view.

Preparations include a clinical assessment of active and passive range of motion of ankle, knee, and hip joints. Key muscles will be neurologically examined according to Janda [38,39], and patients will be asked to perform a single-leg stance to test for Trendelenburg signs. Next, patients will undergo a motion assessment using the Back Performance Scale [40,41]. Reflective skin markers will be placed on previously defined anatomical landmarks (Fig 3), and surface electrodes for EMG will be placed on selected muscles. During the subsequent biomechanical assessment, participants will complete posture, movement, and overground gait assessments in a non-fatigued and in a fatigued state.

For the measurement of the static spinal alignment, participants will be instructed to stand in a natural upright posture with feet hip-width apart, arms hanging relaxed at their sides. In a next step, patients complete five different motion assessments. For the forward flexion assessment, patients will be instructed to stand in a natural upright posture with feet hip-width apart, arms hanging relaxed at the sides, and slowly bend forward at a self-selected speed until the end of the range of motion is achieved, then return to the upright position with knees extended throughout the task. For the pick-up assessment, patients will be asked to grasp and lift a lightweight object (empty box) from the floor. For the trunk torsion assessment, patients will be asked to hold the same object and slightly rotate their body from side to side with arms extended, without moving their feet. Next, data will be captured in a sequence in which the patient will be asked to sit down on a chair, relax for a few seconds, and stand up again. The final movement assessment is called lateral bending, in which the patient will stand in an upright position and will be instructed to bend laterally, while reaching for the knee. For the overground gait analysis, patients will walk back and forth on a walkway at their preferred walking speed.

**Fig 4:**
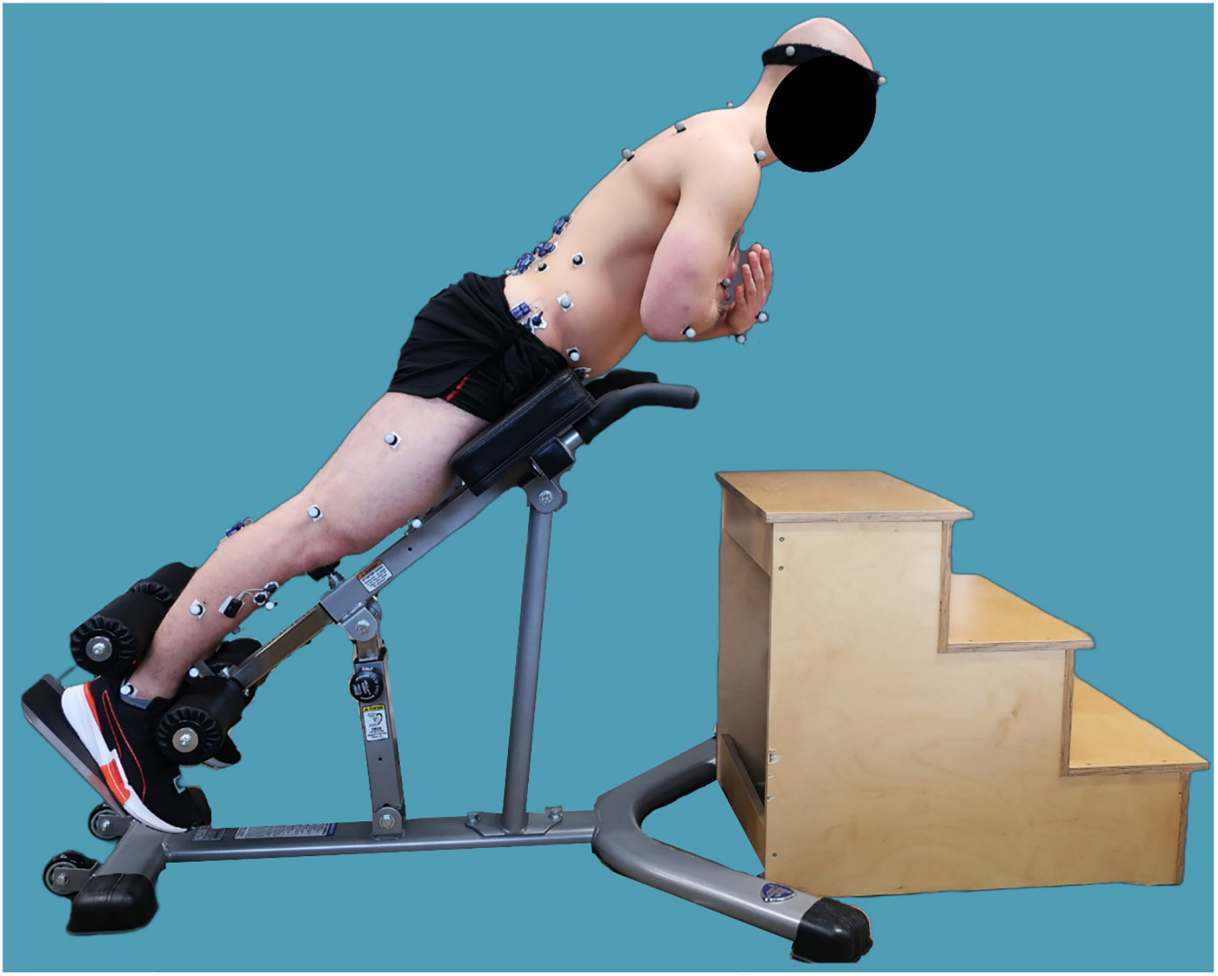
Setup for the modified Biering-Sørensen test.

Paraspinal muscle fatigue will be induced with a modified Biering-Sørensen test [42]. A modified version of the test will be used as we deemed the horizontal trunk position (trunk in line with pelvis and legs) of the standard Biering-Sørensen test to be too demanding for patients with sLSS. We will therefore use a Roman chair (45° inclination, trunk in line with pelvis and legs) in our modified version (Fig 4; approximately 70% demand of standard horizontal position; torque_lumbopelvic_,_modified_ = cos(45°)* torque_lumbopelvic,original_ [43]). The test will be only performed within tolerable pain levels and until the participant decides to support the torso with hands and arms. The duration of the fatigue exercise from start to termination will be measured. To observe the effects of paraspinal muscle fatigue on posture, movement, and gait, the assessments will be repeated immediately after the modified Biering-Sørensen test. Finally, at the end of study visits 1 and 2, participants will be given activity monitors to wear on their wrist for 9 consecutive days.

For the 6-month follow-up, activity monitors will be mailed to participants. Participants will be asked to wear the activity monitor for 9 days and to complete the same digital PROMs.

### Outcome assessment

#### 3D joint kinematics & kinetics

Marker data will be recorded at 120 Hz using a 3-dimensional motion capture system with 10 infrared cameras (Vicon Vero 2.2, Vicon Motion Systems Ltd, Oxford, UK). The set of reflective markers consists of 71 skin markers, including a full-body marker set (53 markers) based on the conventional gait model (CGM) 2.3 [44,45], enhanced by a detailed trunk/spine marker set with 19 markers applied over the spinous processes of the following vertebrae: C7, T1, T2, T3, T4, T5, T6, T7, T8, T9, T10, T11, T12, L1, L2, L3, L4, L5 and S1 (Fig 3). Ground reaction force (GRF) data will be recorded using two force plates (Kistler force plate 9260AA6, Kistler AG, Winterthur; sampling rate 2400 Hz) embedded in the walkway. Marker and force plate data will be recorded during a static posture assessment, selected movement assessments, and overground walking. Spatio-temporal gait parameters will be computed from the marker and force plate data. Joint kinematic and kinetic trajectories will be computed using pyCGM2.3 [44,45], normalized to gait cycle, and peak values and ranges will be computed for each setting, leg, and participant.

#### Electromyographic data

EMG data will be collected during all static and movement trials using a 16-channel EMG system (myon AG, Schwarzenberg, Switzerland, sampling rate 2400 Hz). EMG surface electrodes will be placed bilaterally on the erector spinae (longissimus), erector spinae (iliocostalis), multifidus, gluteus medius, vastus medialis, tibialis anterior and gastrocnemius medialis muscles following the guidelines of the SENIAM project (Surface ElectroMyoGraphy for the Non-Invasive Assessment of Muscles) [46].

#### Patient reported outcome measures

Disability related to sLSS will be assessed with the ODI, Swiss Spinal Stenosis questionnaire and Spine Tango Core Outcome Measures Index for the back (COMI-back). Both the ODI and the Swiss Spinal Stenosis questionnaire are appropriate outcome measures for the treatment of sLSS [47]. The ODI is a self-administered questionnaire consisting of ten items that quantify a patient’s perceived level of functional disability and is considered the gold standard of low back functional outcome tools [31]. In this study, the validated German version of the ODI [30] will be used. The Swiss Spinal Stenosis Questionnaire assesses the intensity of symptoms, physical function, and patient satisfaction following treatment [33]. Spine Tango is the international spine registry for quality control and analysis of outcomes of surgical and non-surgical procedures. The COMI-back is the preferred instrument used in the international spine registry Spine Tango of the European Spine Society. It is a short instrument designed to measure the multidimensional effects of back disorders by evaluating the key patient outcomes (pain, function, symptom-specific well-being, quality of life, disability) [37]. Quality of life and overall health will be assessed with the EQ-5D-5L [32]. It includes five questions/dimensions (mobility, self-care, usual activities, pain, depression) with Likert scales and a visual analogue scale asking patients to rate their general health status from 0 (worst imaginable health status) to 100 (best imaginable health status). The final EQ index is a number between 0 and 1, with 0 indicating the worst possible health status and 1 indicating the best possible health status. Kinesiophobia will be assessed using the Tampa scale of Kinesiophobia [34–36], a 17-item self-report questionnaire based on ratings of fear of exercise, fear of physical activity, and fear avoidance. It consists of two subscales. While activity avoidance focuses on reflection of activities that may increase pain or cause injury, somatic focus examines reflection of beliefs and underlying serious conditions.

#### Spinopelvic and sagittal spinal balance assessed using EOS

**Fig 5:**
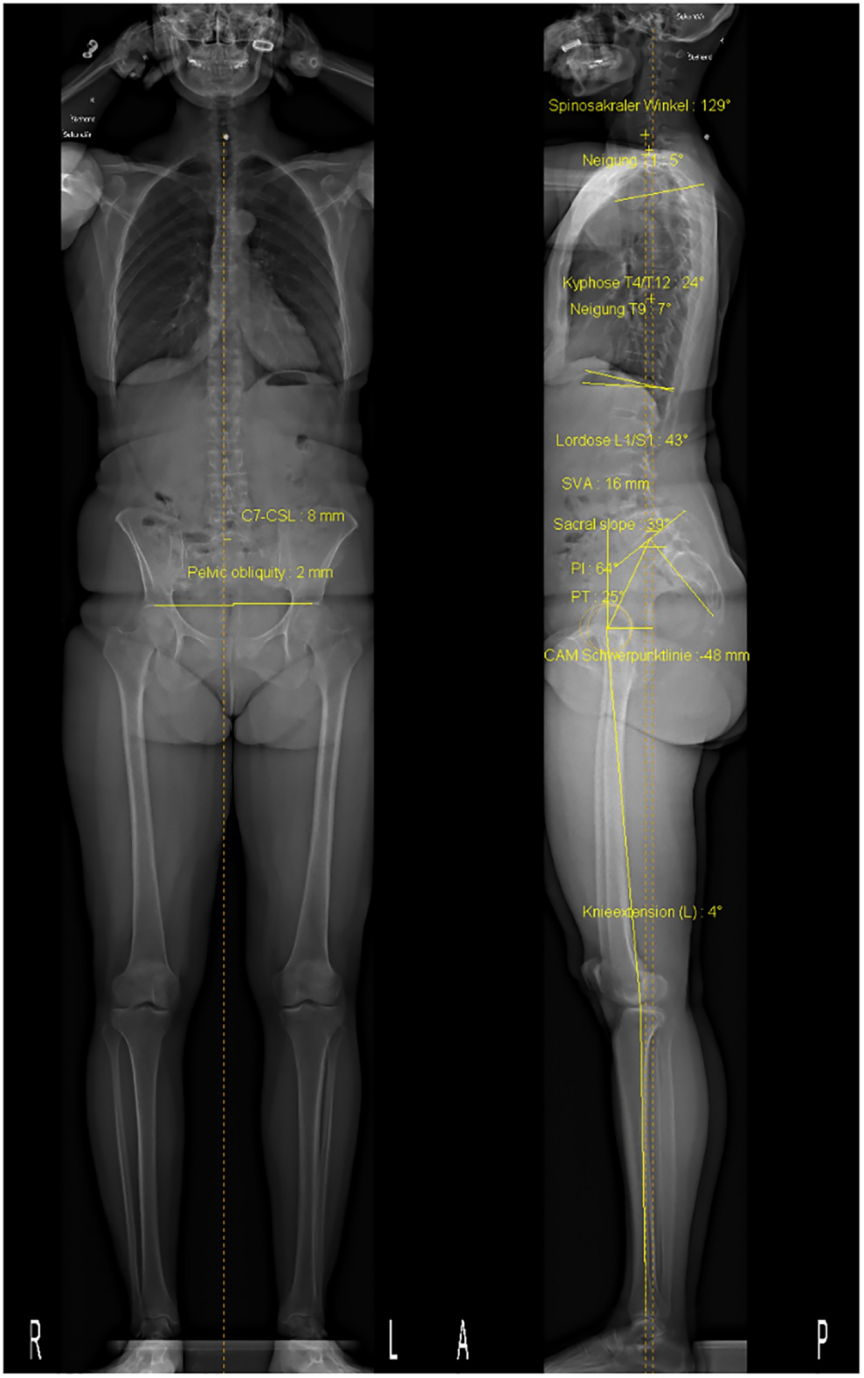
Example of an EOS radiography (left: frontal plane; right: sagittal plane) with measures provided by the sterEOS software.

Radiological spinopelvic and sagittal spinal balance parameters will be obtained using the EOS system (EOS imaging Inc, Cambridge, Massachusetts, USA). The parameters will be calculated semi-automatically using the sterEOS software. Trained radiology technicians follow a workflow in which anatomical landmarks, such as the upper endplate of a particular vertebra, are marked. Once all required landmarks are marked, the established pelvic and sagittal spinal balance parameters are calculated automatically by the sterEOS software: pelvic tilt (PT), pelvic incidence (PI), sacral slope (SS), pelvic obliquity, pelvic rotation, sagittal vertical axis (SVA), spino-sacral angle (SSA), lumbar lordosis (LL) and thoracic kyphosis (TK) (Fig 5) [48]. LL is calculated as the angle between the tangents at the superior L1 and inferior L5 vertebral endplates. Similarly, TK is calculated as the angle between the tangents at the superior T4 and inferior T12 vertebral endplates. SVA is measured as the horizontal distance between the C7 plumb line and the posterior-superior corner of the S1 vertebra. SSA is defined as the angle between the line connecting the centre of the C7 vertebra and the centre of the S1 endplate and the line parallel to the superior S1 endplate. As a quality control measure, all images will be double rated. If two ratings differ by more than 5°, a third radiological technician will rate the images. The final report used for analysis is sent to the patient archive system of the University Hospital Basel.

#### Sagittal spinal balance assessed using motion capture

**Fig 6:**
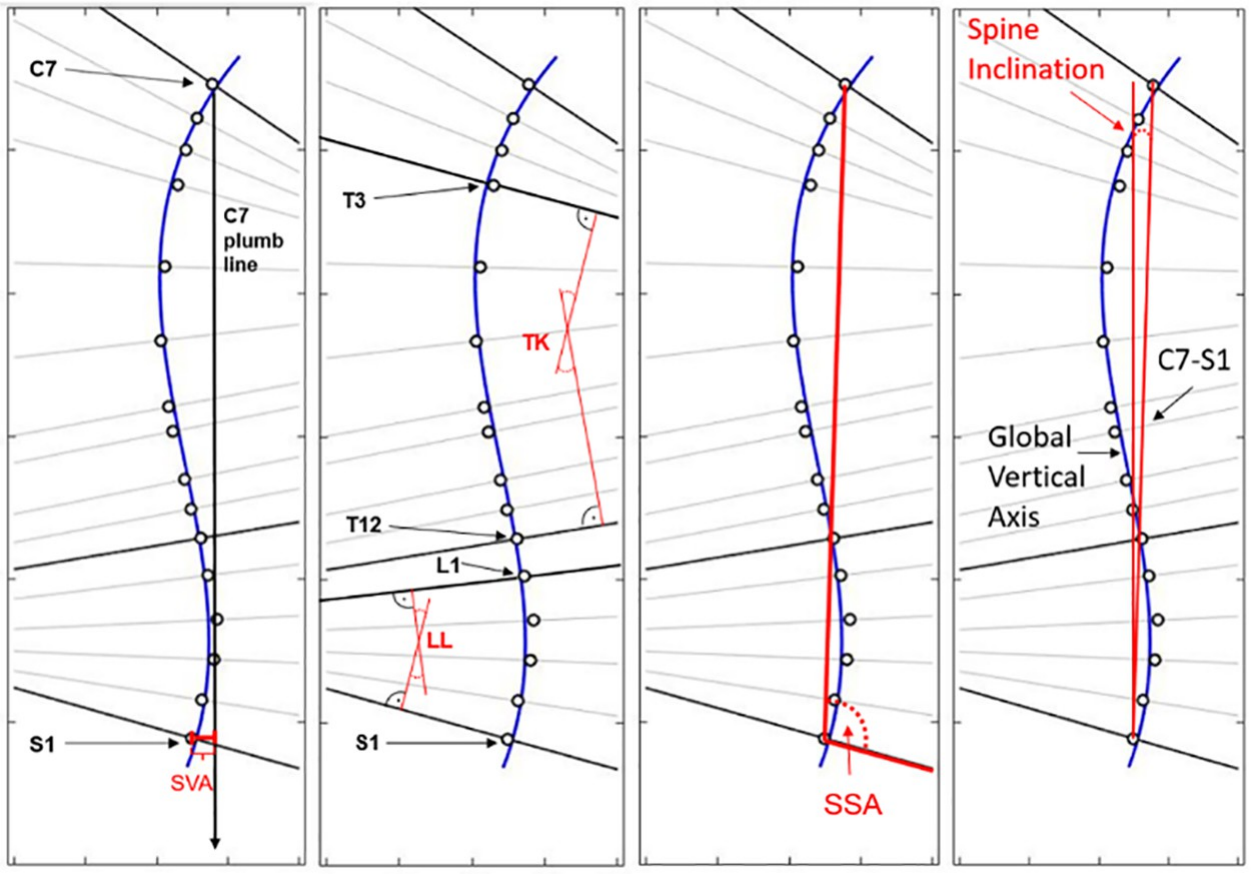
Marker positions (sagittal plane) and calculation of sagittal spinal balance parameters [49] SVA–sagittal vertical axis; LL–lumbar lordosis; TK–thoracal kyphosis; SSA–spino-sacral angle.

The curvature of the lumbar and thoracic spine during standing and walking will be computed from the marker data using MATLAB^™^ (R2022b, The Mathworks Inc., Massachusetts, USA). A cubic polynomial function will be fitted to the marker positions in each time frame to approximate an S-shaped spinal curvature with TK and LL curves [49]. The method for calculating sagittal spinal balance parameters from marker data is based on the calculation methods for radiological sagittal spinal balance parameters and was established and tested in a previous pilot study (EKNZ Project ID 2021–02012) [50,51]. The set of marker-based sagittal spinal balance parameters includes LL, TK, sagittal vertical axis (SVA), spino-sacral angle (SSA), and spine inclination (SI) (Fig 6).

#### Dynamic compensation

Dynamic compensation is defined as the difference between static sagittal spinal alignment during stance and dynamic sagittal spinal alignment during a predefined gait event. Dynamic sagittal spinal balance can be defined as sagittal spinal balance during left/right midstance, left/right heel-strike and/or left/right toe-off. Depending on the research question, we will adapt the workflow and use the most appropriate gait event to calculate dynamic sagittal spinal balance.

#### Sagittal spinal alignment assessed using the optically captured 3D back surface (Artec EVA)

**Fig 7:**
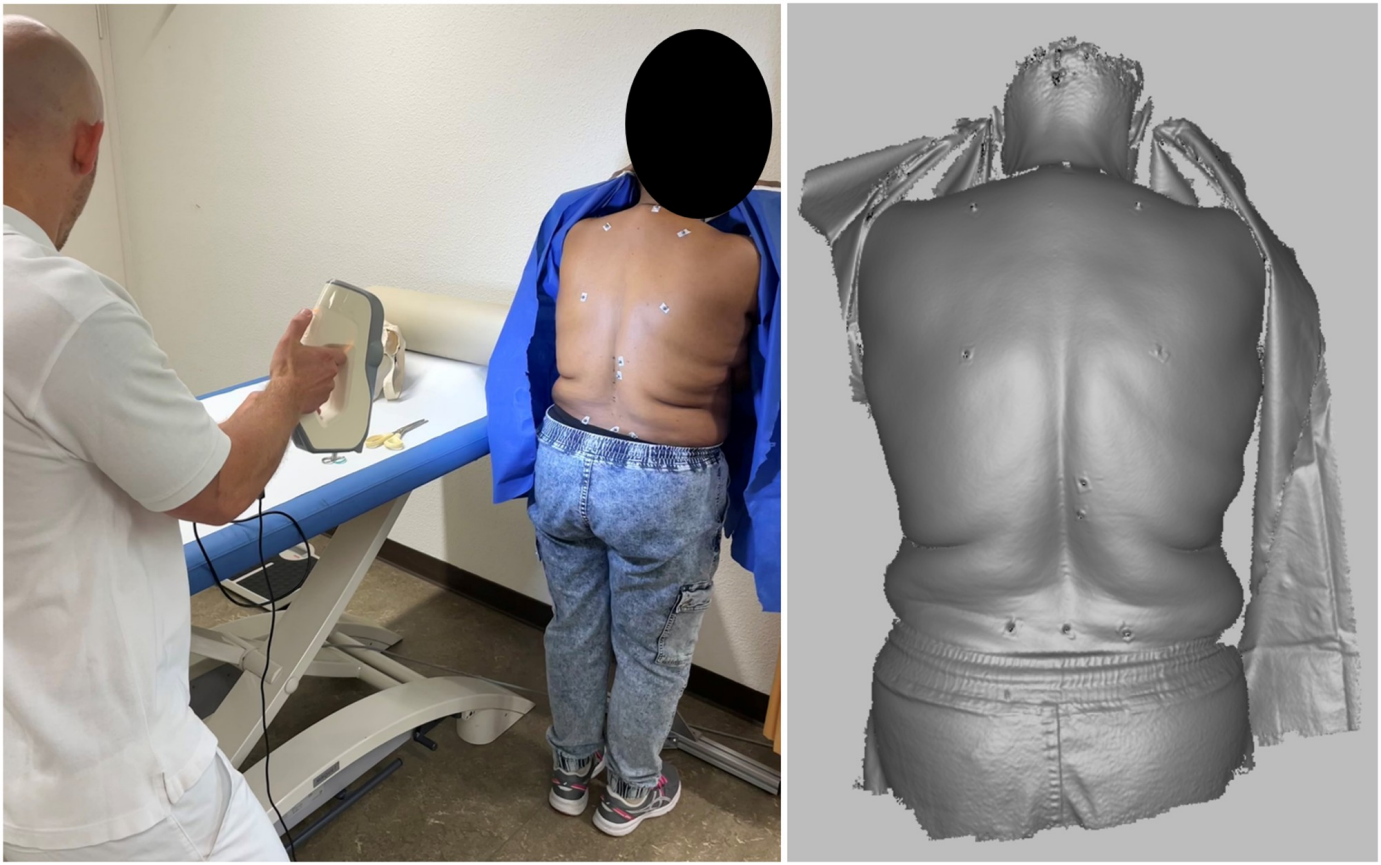
3D optical digitalization with Artec EVA, https://www.artec3d.com, process (left), software output (right).

Artec EVA is a CE-approved optical device used in many human studies for digital reconstruction of surface topography (Artec Europe S.à r.L, Luxembourg, Luxembourg). The device provides a fast, touch- and radiation free technique for scanning the surface of the body. The scanner creates a 3D CAD surface model which can be evaluated directly on a computer screen and requires no additional preparation of the patient (Fig 7). Topography analysis of the surface can reveal asymmetries of the back shape and underlying structures, but also allows calculation of various external postural parameters such as sagittal spinal balance and trunk inclination, as well as kyphotic, lordotic and cervical angles of the back. Recent advances in the study of spinal deformities with noninvasive technologies showed a promising result for mild to moderate cases in patients with adolescent idiopathic scoliosis [52]. The use of such technology in this study may lead to important conclusions about the status of the external shape and postures of patients with sLSS, comparable to standard EOS images. To achieve a better match between the digital surface and the corresponding radiographs, a pose frame (aluminum structure) will be used to reference the posture (elbows) of the subject in both optical and radiological acquisition. The optical surface models will be evaluated together with the EOS images at the ETH Zurich.

#### Muscle fatigue

Muscle fatigue will be quantified using two parameters. First, the duration of the modified Biering-Sørensen test will be measured with a stopwatch. Time will be stopped from the time the patients start the exercise by no longer supporting the torso with their hands until the termination of the exercise by supporting the torso. Second, muscle fatigue will be assessed as the decrease in median EMG frequency over the duration of the Biering-Sørensen test [53,54].

#### MRI outcome parameters

MRI images will be obtained using a 0.55 T MAGNETOM Free.max MRI scanner (Siemens Healthcare, Erlangen, Germany). Our MRI protocol will include the following sequences, set up according to conventional clinical protocols: T1, T2 TSE sagittal, T2 TSE transversal, T2 STIR coronal; VIBE 2-point Dixon transversal. To assess paraspinal and abdominal muscle degeneration, we will quantify muscle atrophy and fatty infiltration. The primary sequence acquired for fat/water quantification will be the VIBE Dixon sequence. For L1 to L5, we will measure the cross-sectional area (CSA) of the abdominal and paraspinal muscles on each side, including the multifidus, erector spinae (longissimus and iliocostalis) and transversus abdominis muscles, and the CSA of the vertebral body. The relative CSA (rCSA) will be calculated as the ratio between the CSA of the muscles and the CSA of the vertebral body and is calculated for each level and side. The CSA of the lean muscle in the region of interest will be defined as LeanCSA and measured on each side. The ratio of LeanCSA to paraspinal muscle CSA will be defined as functional CSA (LeanCSA/CSA), presented as % muscle CSA, and calculated for each level and side. Total CSA, rCSA and LeanCSA will be computed as average CSA (aCSA), average rCSA (arCSA) and average LeanCSA (aLeanCSA) across all levels considering the muscle as a single unit for each side [55]. The severity of stenosis will be classified according to Schizas [56]. Segmental instability will be determined as the relative displacement in anteroposterior position of two adjacent segments between the upright standing radiograph and the supine MRI of more than 3 mm [57].

#### Manual testing for muscle strength

Selected muscles of the legs will be assessed according to Janda’s M5/5 strength levels [38,39]. In a seated position: hip flexion / iliopsoas (L2), knee extension / quadriceps femoris (L3). In the supine position: foot extension (lift up) / tibialis anterior (L4), lifting the big toe / extensor hallucis longus (L5), foot extension (press down) / gastrocnemius (S1). To test for Trendelenburg signs [58], the patient will be asked to stand on one leg. If the pelvis drops to the contralateral side, the test is positive and indicates weakness of the gluteus medius muscle, which is primarily innervated by the L5 nerve root.

#### Physical activity level

Physical activity level will be assessed using an activity monitor (GENEActiv Original, Activinsights, Kimbolton, United Kingdom; sampling rate 50 Hz, dimensions 0.043 x 0.040 x 0.013 m) worn on the non-dominant wrist for 9 days at three occasions: (9 days after first study visit 1; 9 days at 6-month follow-up; and 9 days after study visit 2). The GENEActiv activity monitor has been widely used in scientific research and has been shown to be reliable and valid [59]. Physical activity will be computed for the middle 7 days of the 9-day wear-times to assess normal activity. The recorded accelerometer data will be processed in RStudio (RStudio Team (2023) RStudio: Integrated Development for R. RStudio, Inc., Boston, MA URL http://www.rstudio.com/) using the GGIR script [60].

#### Back performance scale

The Back Performance Scale [40] is an assessment consisting of a series of five movement activities that require trunk mobility. It is routinely used by physical therapists to assess patients with back problems. The activities (sock test, pick-up test, roll-up test, fingertip-to-floor test, and lift test) all require mobility in the sagittal-plane and are scored from 0 (can be performed easily) to 3 (hard/limited to perform). The test has good inter-tester agreement and good test-retest reliability [41].

#### Surgical information

Information about the surgical procedure will be extracted from the patient records and surgery reports. Parameters of interest include whether the patient was treated with decompression alone or along with vertebral fusion. Moreover, the number of decompressed and/or instrumented vertebral levels will be recorded. In the case of instrumentation, we will consider fusions with or without cage. Depending on the patient’s treatment, the cohort may be divided into sub-cohorts.

### Data management

All study data will be entered into and managed using REDCap (Research Electronic Data Capture) hosted at our clinic [61,62]. REDCap is a secure, web-based software platform designed to support data capture for research studies.

### Statistical analysis

Statistical analyses will be performed using RStudio (RStudio Team (2023) RStudio: Integrated Development for R. RStudio, Inc., Boston, MA URL http://www.rstudio.com/). Baseline variables will be reported using mean and standard deviation or median and interquartile range for continuous variables and as counts and percentages for categorical variables. For Specific Aim 1, the primary outcome parameter is the ODI, and the secondary outcome parameter is dynamic compensation (Table 2). Correlation analysis will be used to analyse the relationship between PROMs and continuous variables, including dynamic compensation, spinal alignment and imbalance, segmental instability, muscle degeneration and fatigue, and biomechanical parameters. To measure the association between categorical variables (e.g., stenosis severity) and PROMs, we will perform an analysis of variance (ANOVA).

**Table 2:** Primary and secondary outcome parameters for Specific Aims 1 and 2.

	Primary outcome parameter	Secondary outcome parameter	Additional outcome parameters
Specific Aim 1	ODI	Dynamic compensation	Sagittal spinal alignment assessed using motion capture, EOS and optically captured 3D back surface Patient-reported outcome (Swiss spinal stenosis score, EQ-5D-5L, Tampa score, COMI-back) Muscle fatigue (EMG, fatigue exercise duration) Spatiotemporal gait parameters including joint kinematics/kinetics Muscle atrophy, fatty infiltration, muscle CSA Severity of stenosis Age
Specific Aim 2	Change in pre-to postoperative ODI	Change in pre-to postoperative dynamic compensation	Change in pre-to postoperative… sagittal spinal alignment assessed using motion capture, EOS and optically captured 3D back surface physical activity level patient-reported outcome (Swiss spinal stenosis score, EQ-5D-5L, Tampa score, COMI-back) muscle fatigue (EMG, fatigue exercise duration) spatiotemporal gait parameters including joint kinematics/kinetics muscle atrophy, fatty infiltration, muscle CSA severity of stenosis

ODI–Oswestry disability index; COMI-back–core outcome measures index for the back; EMG–electromyography; CSA: Cross sectional area.

For Specific Aim 2, the primary outcome parameter is the pre- to postoperative change in the ODI, and the secondary outcome parameter is the pre- to postoperative change in dynamic compensation. Pre- to postoperative differences in PROMs, dynamic compensation, severity of stenosis, spinal alignment and imbalance, muscle degeneration, muscle fatigue, and biomechanical parameters will be assessed by comparing means using paired t-tests for continuous variables and by comparing frequencies using the chi-square test for the categorical variables. Associations of pre- to postoperative changes in PROMs with pre- to postoperative changes in static and dynamic spinal imbalance, muscle fatigue, and extent of trunk bending and lumbopelvic range of motion during walking will be assessed by computing the pre- to postoperative difference for each variable for each patient and evaluating the correlation between these computed differences. In the statistical methods described here, a normal distribution of all parameters is assumed. If this assumption is violated in the data, we will use more appropriate statistical methods (e.g., nonparametric methods).

### Sample size calculation

We expect that about half of our patients will present with segmental instability. To assess the correlation between PROMS and continuous covariates, a sample size of 83 is required to detect a correlation of 0.3 at 80% power and 5% significance level. To assess the correlation between PROMS and categorical covariates with 3 levels of the covariate a sample size of 111 (37 within each level) is required to detect a correlation of 0.3 at 80% power and 5% significance level. We hypothesize that the postoperative change in the mean of PROMS at the 12-month follow-up with other covariates is 20%. Between PROMS and continuous covariates a sample size of 34 will be required to detect the difference in mean of 20% with a standard deviation of 40% (effect size = 0.5) and a power of 80% at 5% significance level. Between PROMS and categorical covariates a sample size of 107 will be required to detect the difference in mean of 20% with a standard deviation of 40% (effect size = 0.5) and a power of 80% at 5% significance level. By considering the highest number needed for the study hypothesis, we need 111 patients in this study. Considering a 10% drop-out rate, 122 subjects will be enrolled in the study.

## Discussion

The urgent need for a better understanding of sLSS stems both from the profound socioeconomic impact of the disease on our healthcare system and from the aspiration to provide the best possible treatment to symptomatic patients. The enormous socioeconomic burden this disease places on our society is reflected in the increasing number of symptomatic patients receiving surgery. Today, sLSS is the most frequent indication for spine surgery in people over 65 years of age [10]. The main reason for the increased number of surgeries is due to demographic developments of our society and the fact that individuals aspire higher level of physical functioning in old age. While the prevalence of acquired LSS is controversially discussed and difficult to quantify due to the large number of unreported cases, a recent systematic review and meta-analysis reported a mean prevalence based on radiological diagnosis between 11% and 38% [63]. As shown by the Framingham study [64] and by Ishimoto and colleagues [65], the prevalence both of LSS and sLSS increases with age. Ishimoto et al. observed a prevalence of sLSS in a population resembling the general Japanese population of approximately 10% [65]. With more than 1.6 million persons over the age of 65 in Switzerland [66], up to 600,000 individuals may have an asymptomatic radiologically narrowed lumbar spinal canal, of whom up to 160,000 people currently experience signs and symptoms of sLSS. With the continuing trend of a further aging society, an increasing proportion of persons with an asymptomatic narrow spinal canal can be expected to become symptomatic and require treatment at some point.

Although the number of patients requiring treatment is steadily increasing, there is a lack of studies addressing important questions. For instance, little is known about the association between clinical, radiological, functional, and patient-reported outcomes. To date, it is unclear why not all patients benefit from surgery [67] and why some patients with sLSS remain clinically stable while others will either improve (rarely) or worsen (most likely) over time [68].

The experimental protocol used in this study and the resulting holistic biomechanical, clinical, and functional data will contribute to filling these gaps by contrasting and correlating these aspects. The clinical examinations performed as part of this study, such as the Janda manual muscle strength assessment, or the test for Trendelenburg signs, can be used to relate weakness in certain muscles to objective functional parameters such as gait patterns. Together with the Back Performance Scale, which pragmatically quantifies the level of disability of our patient group during daily activities, the clinical examinations used in this study complement our approach towards a patient-specific analysis.

The selection of PROMs questionnaires is also in line with our aim to obtain a picture as complete as possible. To develop a more accurate picture of patient-reported pain and symptoms related to the stenosis, we decided to assess back symptoms with three established relevant questionnaires: the ODI, the Swiss Spinal Stenosis score, and the COMI-back. While we expect a high correlation among the scores of these three questionnaires, each questionnaire can also be used to cross-validate the other questionnaires. In addition, we use the Tampa score and the EQ-5D-5L questionnaire to assess exercise and movement anxiety, depression, and general quality of life.

One of our previous prospective studies [17] with elective surgery investigated the association between functional limitations (ODI) and gait performance (distance of 6-minute walk test (6MWT) and quality (spatio-temporal parameters and gait asymmetry) in patients with sLSS. Specifically, the ODI decreased by 17.9% and 23.9%, and the walked distance during 6MWT increased by 21 m and 26 m from baseline to 10-week and 12-month follow-up, respectively. Gait quality did not change during the 6MWT at any assessment or between assessments. Compared with the control group, patients walked a shorter distance during the 6MWT, and gait quality differed between patients and the control group at baseline and at 10-week follow-up but not at 12-month follow-up. Change in gait quality explained 39% and 73% of variance in change in ODI from baseline to 10-week and 12-month follow-up, respectively. These results suggest that objective quantitative descriptors of function during daily activity are relevant to perceived disability and are responsive to changes elicited by surgical intervention in patients with LSS.

Among others, this study will provide novel evidence for a possible association between changes in PROMs and changes in physical activity level assessed using GENEActiv. The *in vivo* biomechanical experiment includes a comparison of posture (including spinal curvature) and gait of the patients before and after a muscle fatigue test. Two aspects are of interest to us. First, we are aiming to determine if there is a difference in spinal balance between the static condition during stance and the dynamic condition during gait. If such differences are present, we are interested in the magnitude of this difference, which we refer to as dynamic compensation. Results of our previous pilot study with 30 participants [50, 51] suggest that dynamic compensation occurs in young controls, old controls, and patients with sLSS and is age-dependent. The larger cohort of this study will provide the statistical power to draw conclusions about the association between dynamic compensation, muscle degeneration, severity of stenosis, and PROMs.

In a second step, the relationships between posture, gait, paraspinal muscle fatigue, and paraspinal muscle endurance will be examined to determine whether posture is altered by paraspinal muscle fatigue. To elicit paraspinal muscle fatigue, we chose to use and modify the Biering-Sørensen test (45° Roman chair, extended trunk in line with legs) to reduce the difficulty and increase compliance of patients with sLSS scheduled for surgery. The experiment and the modified Biering-Sørensen test will help to deepen our understanding of different postural compensation strategies and their relationship with paraspinal muscle fatigue.

The collection of EOS radiographs will provide us with high quality data on the spinal balance of each of our participants’ spines and the corresponding spinopelvic alignment. Furthermore, postoperative measurements will provide information on whether spinal imbalance has changed because of the surgical intervention. Finally, radiopaque markers placed on the spine during the radiograph can be used to verify that the markers used in motion analysis are in the correct location.

There are several reasons for collecting current MRI images. First, clinically important parameters such as the exact severity of stenosis on all lumbar segments can be read from the images. Other important parameters are muscle CSA and the extent of fat infiltration of muscle tissue (qualitative and quantitative). According to Fortin et al., the morphology and fat infiltration of the multifidus and psoas muscles are related to patients’ functional level and symptoms [13]. In a previous study [69] it was shown that muscle atrophy and fat infiltration are more pronounced in patients with sLSS than in healthy controls. Further, fat infiltration of the lumbar paraspinal muscles is significantly and positively associated with the severity of lumbar degenerative disease [70]. A recent retrospective study [28] of 165 patients investigated the association between fat infiltration of paraspinal muscle, sagittal spinopelvic alignment, and stenosis severity in patients with degenerative LSS. The results suggest that fat infiltration of paraspinal muscles is associated with sagittal spinopelvic alignment, which does not appear to be associated with the natural aging process. Patients with more severe fat infiltration had greater sagittal spinopelvic alignment mismatch. This association appears to be specific to patients with degenerative spinal deformity, as it was not present in asymptomatic adult persons [71,72]. Overall, these results suggest that fat infiltration of paraspinal muscles might play an important role in LSS. Because our MRI protocol includes axial VIBE Dixon sequences with an additional coil on the abdomen, we can assess and quantify the fat fraction and CSA of not only paraspinal but also abdominal muscles, such as the transversus abdominis. Together with the multifidus and the pelvic floor muscles, the transversus abdominis forms the anatomical girdle that is critical for providing spinal stabilization [73,74]. Investigating the relationship between abdominal and paraspinal muscles will provide additional insights into the pathophysiology of LSS.

Together with the actual anatomical reference values from the standing EOS radiography, and the information regarding the muscle quality and muscle degeneration from the MRI, the unique data set obtained in this study will be useful for driving state-of-the-art *in silico* musculoskeletal models with the goal to simulate spinal loads at each vertebral level.

Finding a correlation between PROMs, spinal imbalance, muscle degeneration, dynamic compensation, muscle fatigue, severity of stenosis, and biomechanical parameters would confirm the implicit rationale regarding the assumed clinical relevance of these factors. A correlation between pre- to postoperative changes in PROMs and pre- to postoperative changes in spinal alignment, muscle fatigue and fatty degeneration, and biomechanical parameters would confirm the hypothesis that surgery influences parameters that determine patient-reported outcome. The results of these experiments may help to develop new strategies for both conservative as well as surgical management of patients with sLSS. For instance, specific muscle strengthening exercises or specific stabilization approaches might be employed that consider the altered biomechanics of the spine. Finally, the results of this study might serve as a basis for developing an algorithm to predict surgical outcome. Being able to predict surgical outcome would be a critical added value to patient care and translate in improved patient satisfaction while lowering health care costs.

## Supporting information

S1 Checklist SPIRIT 2013 checklist: Recommended items to address in a clinical trial protocol and related documents*. (PDF)

S1 File First approved study protocol by EKNZ. (PDF)

S2 File First approval of study protocol by EKNZ. (PDF)

S3 File Last amendment of study protocol to EKNZ. (PDF)

S4 File Approval of last amendment of study protocol by EKNZ. (PDF)

S5 File Confirmation of approval. (PDF)

S6 File Confirmation of funding. (PDF)
