# Supplementary material for: The role of muscle degeneration and spinal balance in the pathophysiology of lumbar spinal stenosis: Study protocol of a translational approach combining in vivo biomechanical experiments with clinical and radiological parameters
Source: PLoS One. 2023 Oct 27;18(10):e0293435. doi: 10.1371/journal.pone.0293435 (PMC10610482; doi:10.1371/journal.pone.0293435)
Supplement: S1 File — (PDF) [file pone.0293435.s002.pdf]

Swiss National Science Foundation

Unit Projects Life Sciences
Wildhainweg 3, P.O. Box
3001 Bern

+41 31 308 22 22
projects.ls@snf.ch
www.snsf.ch

To whom it may concern

Berne, 18 September 2023

320030_204461

The Swiss National Science Foundation SNSF is the main public funding agency for academic research in Switzerland.
We herewith confirm that the study protocol for the project 320030_204461 " RoLSSroice - Role of spinal load in the pathophysiology of lumbar spinal stenosis: a translational approach combining clinical, functional and radiological parameters, in vivo biomechanical experiments and advanced in silico musculoskeletal modeling" was selected for funding after independent peer review. The SNSF has no self-interest in the study outcomes.

Yours sincerely

Christoph Meier, PhD
Head of Unit Project Life Sciences
