# Supplementary material for: The role of muscle degeneration and spinal balance in the pathophysiology of lumbar spinal stenosis: Study protocol of a translational approach combining in vivo biomechanical experiments with clinical and radiological parameters
Source: PLoS One. 2023 Oct 27;18(10):e0293435. doi: 10.1371/journal.pone.0293435 (PMC10610482; doi:10.1371/journal.pone.0293435)
Supplement: S2 File — (PDF) [file pone.0293435.s003.pdf]

[redacted]

RoLSSroice – Role of spinal load in the pathophysiology of lumbar spinal stenosis: A translational approach combining clinical and radiological parameters, in vivo biomechanical experiments and advanced in silico musculoskeletal modeling

Research legislation:	Ordinance on human research with the exception of Clinical trials (HRO) [1].
Type of Research Project:	Research project involving human subjects
Risk Categorisation:	Risk category A
Project Leader:	PD Dr. med. Cordula Netzer<br>Deputy Head, Spine Center<br>Department of Spine Surgery<br>University Hospital Basel<br>Spitalstrasse 21<br>CH-4031 Basel<br>Phone +41 61 265 78 30<br>Fax +41 61 265 70 09<br>cordula.netzer@usb.ch

PROTOCOL SIGNATURE FORM

Study Title	***RoLSSroice – Role of spinal load in the pathophysiology of lumbar spinal stenosis: A translational approach combining clinical and radiological parameters, in vivo biomechanical experiments and advanced in silico musculoskeletal modeling***

The project leader has approved the protocol version 02 *(dated 05.08.2022)* and confirms hereby to conduct the project according to the protocol, the Swiss legal requirements [1, 2], current version of the World Medical Association Declaration of Helsinki [5] and the principles and procedures for integrity in scientific research involving human beings.

Project leader:

Name: PD Dr. med. Cordula Netzer

Date: 05.08.2022 Signature: [redacted]

Sponsor:

University Hospital Basel, Spine Surgery

Name: Prof. Dr. med. Stefan Schären

Date: 05.08.2022 Signature: [redacted]

TABLE OF CONTENTS

TABLE OF CONTENTS 3
GLOSSARY OF ABBREVATIONS 4
1. BACKGROUND AND PROJECT RATIONALE 5
2. PROJECT OBJECTIVES AND DESIGN 6
2.1 Hypothesis and primary objective 6
2.2 Primary and secondary endpoints 6
Primary endpoints 6
Secondary endpoints 6
Other parameters 7
2.3 Project design 7
3. PROJECT POPULATION AND STUDY PROCEDURES 8
3.1 Project population, inclusion and exclusion criteria 8
3.2 Recruitment, screening and informed consent procedure 8
3.3 Study procedures 8
3.4 Project plan 14
3.5 Withdrawal and discontinuation 14
4 STATISTICS AND METHODOLOGY 14
4.1 Sample size calculation 14
4.2. Statistical analysis plan 15
4.3. Handling of missing data 15
5 REGULATORY ASPECTS AND SAFETY 15
5.1 Local regulations / Declaration of Helsinki 15
5.2 Notification of safety and protective measures (HRA Art. 15, HRO Art. 20) 16
5.3 Serious events (HRO Art. 21) 16
5.4 Procedure for investigations involving radiation sources 16
5.5 Amendments 16
5.6 End of project 16
5.7 Insurance 16
6 FURTHER ASPECTS 16
6.1 Overall ethical considerations 16
6.2 Risk-Benefit Assessment 17
7 QUALITY CONTROL AND DATA PROTECTION 17
7.1 Quality measures 17
7.2 Data recording and source data 17
7.3 Confidentiality and coding 17
7.4 Retention and destruction of study data and biological material 18
8. FUNDING / PUBLICATION / DECLARATION OF INTEREST 18
9. REFERENCES 19

GLOSSARY OF ABBREVATIONS

aCSA	*average CSA*
arCSA	*average relative CSA*
aLeanCSA	*average LeanCSA*
BASEC	*business administration system for ethical committees*
CSA	*cross-sectional area*
BMI	*body mass index*
CRF	*case report form*
C7PL	*C7 plumb line*
EMG	*electromyography*
FOPH	*federal office of public health*
FJ	*facet joint*
HRA	*human research act*
HRO	*ordinance on human*
LeanCSA	*CSA of lean muscle in region of interest*
LSS	*lumbar spinal stenosis*
LL	*lumbar lordosis*
MRI	*magnetic resonance imaging*
ODI	*Oswestry disability index*
PROMs	*patient-reported outcome measures*
rCSA	*relative cross-sectional area*
SI	*spine inclination*
sLSS	*symptomatic lumbar spinal stenosis*
SSA	*spinosacral angle*
SVA	*sagittal vertical axis*
TK	*thoracic kyphosis*

1. BACKGROUND AND PROJECT RATIONALE

Lumbar spinal stenosis (LSS) is a common syndrome affecting the human spine characterized by age related degeneration of the lumbar discs, facet joints (FJs) and hypertrophy of the ligamentum flavum resulting in pain, limited function and compromised quality of life. Symptomatic LSS (sLSS) is an often highly disabling condition [6], the most common reason for spinal surgery in patients older than 65 years of age [7, 8] and represents a major financial burden to the health care system [9]. sLSS has been associated with disability [10], atrophy and fatty infiltration of paraspinal muscles [11], decreased physical activity [12], walking capacity [10], gait patterns and stability [13-15], balance [16] and alterations of the sagittal balance of the spine [17]. However, the natural course and the pathophysiological processes of LSS are not yet fully understood.

The spine's overall function is determined by the interrelationship between the pelvis, the sacrum and the local lumbar, thoracic and cervical spinal curvatures. In a healthy spine, the local curvatures result in a physiological alignment requiring minimal muscular activity for maintenance of upright stance. This state is called global spinal balance. In this state, global and local spinal loads during static posture and dynamic motion will have minimal effects on the spinal canal. However, spinal balance and loads altered by the presence of sLSS may result in further narrowing of the spinal canal and compression of the neural elements or in overloading of the already degenerated lumbar segments possibly eliciting typical pain symptoms. Clinical observations have shown the presence and intensity of symptoms depending on the body posture (e.g., exacerbated while standing, relieved while sitting or lying) or activity (e.g., exacerbated while walking, and relieved while forward bending) [18]. These observations indicate an influence on function and the relevance of physiological loads. Hence, understanding the interrelationship between spinal load, kinematics and functional disability is one of the key factors in the prevention of this disease. Although these observations clearly show that spinal motion and loading plays a critical role in clinical presentation of lumbar spinal stenosis, to date dynamic *in vivo* loads acting on the spine during activities of daily living in patients with sLSS are largely unknown. As an alternative to invasive *in vivo* measurement of spinal loads, computational modelling methods allow for non-invasive investigation of spinal loads *in silico* [1, 19-21].

In the proposed project, we will assess sagittal spinal balance and motion in patients with sLSS using an optoelectronic method based on infrared cameras and retroreflective markers. We will elicit paraspinal muscle fatigue using a modified version of the Biering-Sørensen test and compare sagittal spinal balance and motion before and after the fatigue exercise, which will allow to associate sLSS-specific motion patterns to paraspinal muscle fatigue. Additional data generated using magnetic resonance imaging (MRI) allows detecting associations between sLSS, muscle degeneration and fatty infiltration. Moreover, we will obtain radiological images from the spine in upright position using EOS, a specialized low-dose x-ray unit. These images will allow the calculation of the anatomical global and local sagittal spinal balance, enabling a characterization of spinal kinematics in patients with sLSS and a validation of the workflow based on the optoelectronic method. Coded data obtained from EOS and motion analysis will allow optimizing existing biomechanical musculoskeletal models of the human spine.

The results of this study will provide first mechanistic evidence of the role of clinical, radiological, functional and biomechanical factors in spine load. The combination of *in vivo* experiments with *in silico* experiments represents a unique opportunity of translating knowledge gained from systematic experiments considering biological measurements back to the patient. Confirming the relationship between clinical, radiological, functional and biomechanical factors and local load will provide the necessary evidence for using the framework employed in this project for diagnostics, treatment and rehabilitation planning in patients with LSS and predicting the outcome of existing and novel therapies. The recommendations based on our results will directly impact the treatment of patients at our clinic by identifying patient-specific parameters responsible for large local loads that will allow targeting therapy specifically at these parameters facilitating patient specific evidence-based medicine and ultimately reduce the risk of recurrences of symptoms and/or stenosis after surgery.

2. PROJECT OBJECTIVES AND DESIGN

2.1 Hypothesis and primary objective

The primary objective of this study is to improve the understanding of the role of postural and ambulatory biomechanics for symptoms in patients with sLSS.

Primary hypothesis

Hypothesis 1: PROMs correlate with dynamic compensation (difference between static and dynamic sagittal spinal alignment) in patients with sLSS.

Secondary hypotheses

Hypothesis 2.1: PROMs correlate more strongly with quantitative functional parameters including muscle fatigue, spinal load and biomechanical parameters or lumbopelvic range of motion during forward trunk bending and during walking than with pathomorphological parameters including severity of stenosis, segmental instability and muscle degeneration.

Hypothesis 2.2: Changes in PROMs correlate with changes in static and dynamic spinal imbalance, muscle fatigue and extent of forward trunk bending, lumbopelvic range of motion during walking and spinal loads from before to after spinal stenosis surgery.

Hypothesis 2.3: Patients with sLSS with greater muscle degeneration and greater spinal imbalance have greater muscle fatigue.

Hypothesis 2.4: At follow-up, patients with spinal stenosis will have improved PROMs, less static and dynamic spinal imbalance and muscle fatigue, and less forward trunk bending and larger lumbopelvic range of motion during walking than before spinal stenosis surgery.

Hypothesis 2.5: Postoperative PROMs are explained by preoperative severity of LSS, spinal alignment, segmental instability, muscle degeneration, dynamic compensation, muscle fatigue and spinal load.

2.2 Primary and secondary endpoints

Primary endpoints

I. Oswestry disability index (ODI)
II. Dynamic compensation

Secondary endpoints

III. Sagittal spinal alignment and spinal loads assessed using motion capture
IV. Sagittal spinal alignment assessed using EOS
V. Muscle fatigue
   - assessed using electromyography (EMG)
   - fatigue exercise duration
VI. Radiological parameters
   - Muscle atrophy
   - Fatty infiltration
   - Muscle cross-sectional area
   - Stenosis severity
   - Segmental instability

Other parameters

VII. Quality of life (EQ-5D-5L)
VIII. Swiss spinal stenosis score
IX. Tampa scale of kinesiophobia
X. Core Outcome Measures Index for the back (COMI back)
XI. Upper body motion (inertial sensor data)
XII. Manual testing for muscle strength
XIII. Physical activity level
XIV. Back performance scale
XV. Surgical information
  - Scheduled decompression surgery
  - Scheduled fusion of vertebrae
  - Number of decompressed levels
  - Number of instrumented levels
  - In case of instrumentation:
    - Fusion with Cage
    - Fusion without Cage

XVI. ICHOM
  - Age
  - Sex
  - Body mass
  - Body height
  - Body mass index (BMI)
  - Pain medication
  - Previous treatment (physiotherapy, infiltration, other)
  - Own physical exercise program
  - Education, occupation, level of permanent invalidity, social situation, need of care
  - Duration of symptoms categorized as <1 year, 2−5 years, and >5 years

2.3 Project design

This study is designed as a single-center, longitudinal observational study. For each patient, a multimodal set of data including experimental, clinical, functional, radiological and biomechanical data is compiled before and after routine surgical intervention. There will be two study visits: baseline and one-year follow-up (Figure 1).

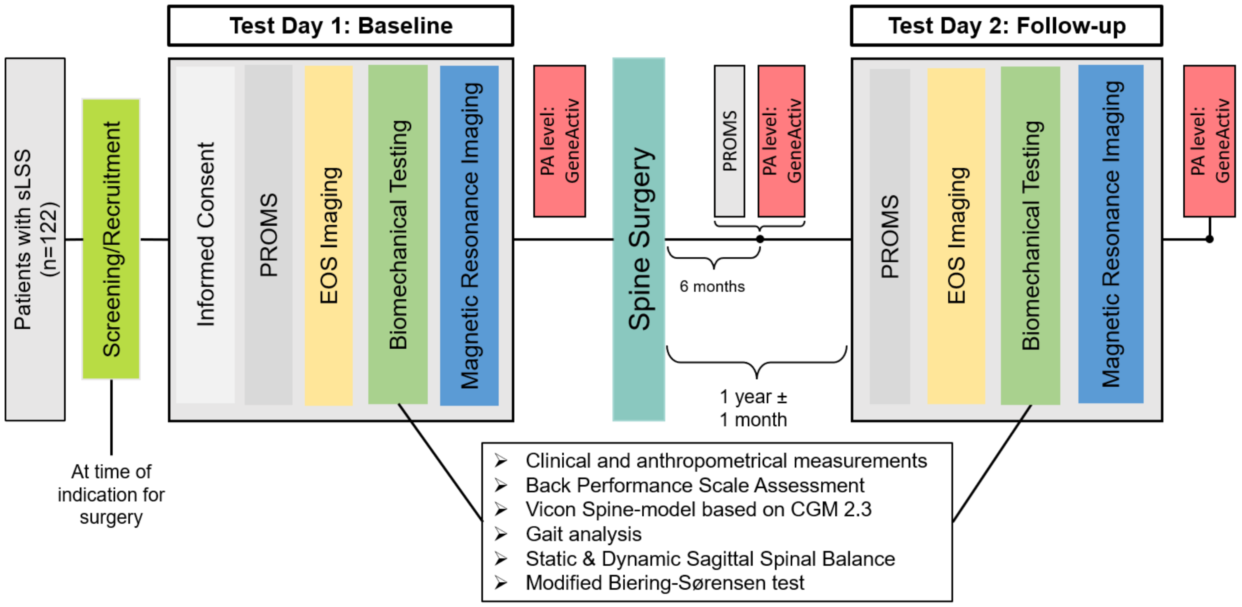

Figure 1: RoLSSroice study flow chart

3. PROJECT POPULATION AND STUDY PROCEDURES

3.1 Project population, inclusion and exclusion criteria

We plan to include 122 patients with sLSS (61 per year) in this study, which will be realistic and sufficient for the proposed statistical analysis (see Section 4.1). Patients will be recruited at the Department of Spine Surgery at the University Hospital Basel. The surgical procedure is not affected by the study and is not subject of investigation. All patients will receive – depending on their clinical and imaging diagnostics – lumbar decompression alone or decompression combined with fusion either through posterolateral fusion with pedicle screw-rod system with autologous bone apposition or through transforaminal lumbar interbody fusion with a cage filled with autologous bone implanted from posterolateral into the intervertebral space. Decompression will be performed as either open surgery or microsurgical technique, and both are performed through a midline approach. The open surgery decompression will be performed via an interspinous approach with interlaminar flavectomy, whereas in the microsurgical technique, uni- or bilateral fenestration and flavectomy will be performed.

Inclusion criteria:

- age > 30 years
- BMI < 35 kg/m^2
- diagnosed sLSS
- clinical symptoms for at least 6 months
- intermittent neurogenic claudication with limitations of their walking ability due to symptoms in the lower back and or in one or both legs
- unsuccessful conservative treatment
- confirmation of the LSS through MRI
- scheduled for surgery

Exclusion criteria

- inability to provide informed consent
- previous spine surgery
- use of walking aids
- other neurologic disorders affecting gait
- MRI incompatibility
- pregnancy

3.2 Recruitment, screening and informed consent procedure

Patients with sLSS will be recruited at the Clinic for Spine Surgery at the University Hospital Basel (Switzerland). Clinical treatment will not be affected by the study. Patients will be informed about the study at the time of indicating for surgery. In addition, patients will be screened using the hospital's surgery schedule tool. Patients with sLSS scheduled for decompression surgery will be selected by the PhD student and/or trained members of the research group. Medical history, inclusion and exclusion criteria of the screened patients will be checked by the study physician. Eligible patients will be contacted and informed about the study. Inclusion- and exclusion criteria will be checked again during the call and at the time of consent.

3.3 Study procedures

After the patients' arrival at the movement laboratory, the patient is informed in detail about all study procedures. If there are no questions from the patient, the informed consent form is signed. Subsequently, clinical and demographic parameters including age, body mass, body height, pain medication, previous treatment (physiotherapy, infiltration, other), duration of symptoms and own physical exercise program will be recorded on the case report file. In a next step, participants will

be asked to complete several PROMs questionnaires including the validated German versions of the ODI, EQ-5D-5L, Swiss Spinal Stenosis Questionnaire, Tampa scale of kinesiophobia and Spine Tango COMI. PROMs will be digitally collected using iPads and surveys created on the REDCap® [22] web application for building and managing online surveys and databases (approximate duration: 15 minutes).

After completion of the questionnaires, all participants will be prepared for the functional biomechanical analysis, which starts with a clinical measurement of the active and passive range of motion of ankle, knee and hip joints. This also includes a neurological examination of the key muscles according to Janda, and a test used to elicit Trendelenburg signs. Next, the back performance scale movement assessment will be performed. The assessment includes five separate tests (Sock Test, Pick-up Test, Roll-up Test, Fingertip-to-Floor Test, and Lift Test) that will only be performed if allowed by the patients' current pain situation (approximate duration: 30 minutes).

Prior to placement of surface EMG electrodes, specific skin areas will be shaved and cleaned, if necessary. Electrodes will be placed on selected abdominal and paraspinal muscles. Reflective markers will be placed on anatomical landmarks (approximate duration: 45 minutes).

Participants will complete forward-bending, pick-up, trunk torsion, sitting and walking assessments before and after a fatigue exercise. Paraspinal muscle fatigue will be elicited by a modified Biering-Sørensen test [23] on a Roman chair (45° chair inclination, trunk horizontal). The modified Biering-Sørensen test will be conducted immediately before the movement assessments will be repeated to obtain data in the fatigued state. At the very end of the movement analysis, patients will complete the sock test from the back performance scale assessment again with the retroreflective markers attached (approximate duration: 30 minutes).

After the functional biomechanical analysis, radiological data (MRI and EOS) will be collected. MR imaging of the lumbar and abdomen region will be performed using a 0.55T MAGNETOM Free.max scanner (Siemens Healthcare, Erlangen, Germany) with the patients in supine position with extended legs. MR imaging allows the classification of level and severity of stenosis. Multi-echo gradient echo (VIBE Dixon) axial images will be acquired for the analysis of the abdominal and paraspinal muscles. A T2-weighted axial image will be selected at each vertebral body level in the center of the body itself identified on the sagittal view image. All sequences are not enhanced (approximate duration: 30 minutes). Finally, upright standing sagittal plane EOS images of the full body including entire spine and pelvis will captured (approximate duration: 10 minutes). The total duration of the baseline study visit on test day 1 including travel times between facilities will be approximately 3.5 hours. Because baseline and 1-year follow-up entail the same study procedures, a complete study participation will take approximately 7 hours.

Participants will be handed out activity monitors after test-day one and test-day two. The same activity monitor will be sent to study participants six months after the spine surgery. Participants will be asked to wear the activity monitor for nine days on their wrist. Participants will be given prepaid envelopes to return the devices. In addition, participants will be asked to complete a digital set of PROMs six months after the spine surgery sent to them via e-mail. If no e-mail address is available, printed versions will be sent together with the activity monitor.

Movement data collection and processing

Biomechanical parameters will be recorded with 120 Hz using a 3-dimensional motion capture system with 10 infrared cameras (Vicon Vero 2.2, Vicon Motion Systems Ltd, Oxford, UK). The marker set will consist of 71 reflective skin markers, including a full-body marker set (53 markers) based on the Conventional Gait Model (CGM) 2.3 [24], enhanced by a self-induced detailed trunk/spine marker set with 19 markers applied over the spinous processes of the following vertebrae: C7, T1, T2, T3, T4, T5, T6, T7, T8, T9, T10, T11, T12, L1, L2, L3, L4, L5 and S1 (Figure 2). The CGM 2.3 requires the measurement of anthropometrical parameters including leg length, knee width, malleolar-width, shoulder offset, elbow width, wrist width and hand thickness.

Patients will wear their normal comfortable flat shoes. Spatio-temporal and kinematic data will be recorded for all movement assessments and gait trials. For the measurement of the static spinal alignment, participants will be instructed to stand in a natural upright posture with their feet hip-wide apart, arms hanging relaxed at the sides. For the forward-bending assessment, patients will be instructed to stand in a natural upright posture with their feet hip-wide apart, arms hanging relaxed at the sides, and slowly bend forward at a self-selected speed until the end of range of motion is achieved and then return to the upright position, while keeping their knees extended during the entire task. For the pick-up assessment, patients are asked to grab and lift an object from the ground and place it on a table (height: 76cm). During the trunk-torsion assessment, patients are asked to grab an object (empty card board box) and move it from one side of the table to the other side with extended arms without moving their feet. Next, data are captured in a sequence during which the patient is asked to sit down on a chair, relax for a few seconds and stand up again. Then, patients will walk back and forth across the walkway at their preferred walking speed.

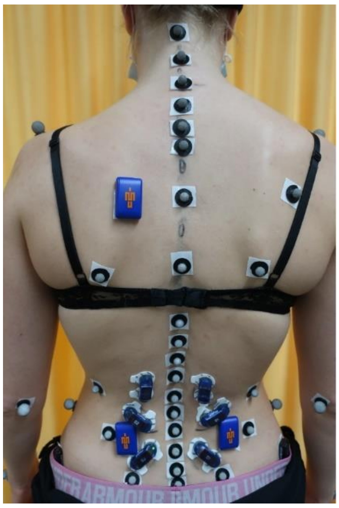

Figure 2: Dorsal Marker Placement

During the fatigue exercise, paraspinal muscle fatigue will be elicited by a modified Biering-Sørensen test [23] on a Roman chair (45° chair inclination, trunk horizontal). The test is only conducted within tolerable pain levels until the participant terminates the exercise by choosing to support the torso with hands and arms on a cushion placed below the trunk. The duration of the fatigue exercise from start until termination will be measured.

Assessment of study parameters

I. Oswestry disability index (ODI)

Disability related to sLSS will be assessed with the ODI, which is considered the gold standard of low back functional outcome tools [25]. The ODI is a questionnaire comprising 10 self-administered items that quantify a patient's perceived level of functional disability. Each of the items relates to a different area of functional impairment and consists of six statements, which are scored from zero to five points. Patients choose the statement that suits their subjective perception best. In this study, the validated German version of the ODI [26] will be used.

II. Dynamic compensation

Dynamic compensation is defined as the difference between static and dynamic sagittal spinal alignment. Six possible gait events are available to choose from for the definition of dynamic sagittal spinal balance (left and right; heel-strike, toe-off, midstance). Dynamic sagittal spinal balance may be defined as sagittal spinal balance during left/right midstance, left/right heel strike and/or left/right toe off. Depending on the research question, we will adapt the workflow and use the most appropriate gait event to calculate dynamic sagittal spinal balance.

III. Sagittal spinal balance and spinal loads assessed using motion capture

The curvature of the lumbar and thoracic spine during stance and during walking will be computed from the marker data using MATLAB™ (R2021b, The Mathworks Inc., Massachussetts, USA) software. A cubic polynomial function will be fitted to the marker positions in each time frame, approximating an S-shaped spine curvature with thoracic kyphosis (TK) and lumbar lordosis (LL) curves [1]. The workflow used to calculate sagittal spinal balance parameters is based on the calculation methods for radiological sagittal spinal balance parameters and was established and tested in a previously conducted pilot study (EKNZ Project ID 2021-02012). The set of marker-based sagittal spinal balance parameters comprises LL, TK, sagittal vertical axis (SVA), spino-sacral angle (SSA), spine inclination (SI) (Figure 3).

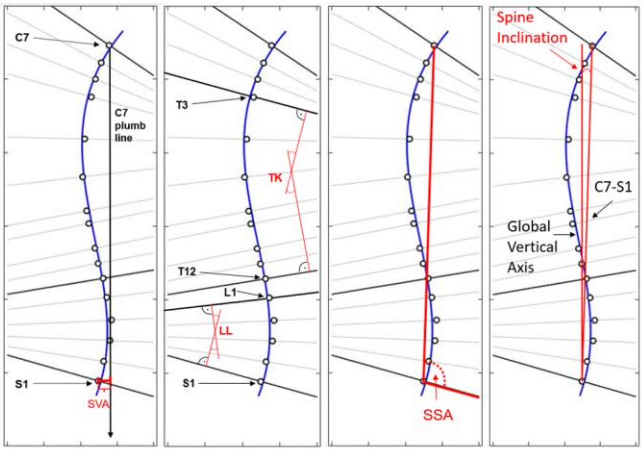

Figure 3: Computed Marker Data and Calculation of Sagittal Spinal Balance Parameters [1]

The data obtained using motion capture will be used to estimate spinal load using musculoskeletal modeling [27].

IV. Sagittal Spinal Balance assessed using EOS

Sagittal spinal balance parameters from EOS radiological images are calculated semi-automatically using the sterEOS software provided by the EOS company. Trained radiology technicians follow a workflow during which anatomical landmarks, e.g., the center of a specific vertebra, are marked in the EOS software. Once all required landmarks are marked, the parameters are calculated automatically by the sterEOS software.

This workflow allows the calculation of four sagittal spinal balance parameters: SVA (Figure 4A), SSA (Figure 4A) LL (Figure 4B) and TK (Figure 4B) [28]. LL is calculated as the angle between the tangents on the superior L1 and inferior L5 vertebral endplates. Likewise, TK is calculated as the angle between the tangents on the superior T4 and inferior T12 vertebral endplates. SVA is measured as the horizontal distance between C7 plumb line and the posterior-superior corner of the S1 vertebra. SSA is defined as the angle between the line connecting the center of the C7 vertebra to the center of the S1 endplate and the line parallel to the superior S1 endplate. As a quality control measure, all images are double rated. If two ratings are apart more than five degrees, a third radiological technician rates the images. The final report, which will be used for the analysis, is sent to the patient archive system of the University Hospital Basel.

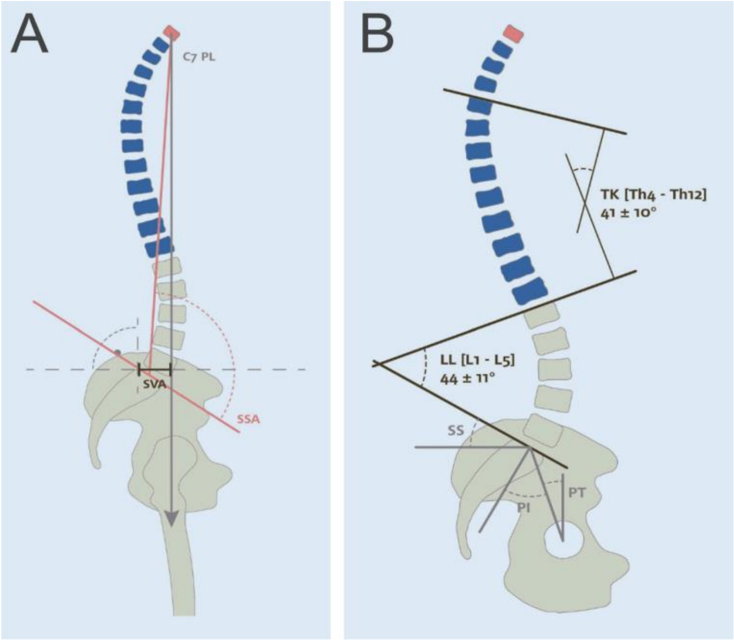

Figure 4: Overview of Spinopelvic Sagittal Alignment Parameters (modified from Völlner and Grifka [2]

V. Muscle fatigue

Muscle fatigue will be quantified using two parameters. First, the fatigue exercise duration will be measured using a stopwatch. Time is stopped from the point where the patients starts the exercise by no longer supporting the torso with their hands until the termination of the exercise by supporting the torso. Second, muscle fatigue will be assessed as the decrease in median EMG frequency as described by Boutellier et al. (2021) [29].

VI. Radiological parameters

The measurement of *muscle atrophy* and *fatty infiltration* allows quantifying abdominal and paraspinal muscle degeneration. The analysis will be performed using ImageJ image analysis software (Version 1.52t, National Institutes of Health, Bethesda, Maryland) [30, 31].

For L1 to L5, we will measure the *cross-sectional area* (CSA) of the *abdominal and paraspinal muscles* on each side, including the multifidus and the erector spinae (longissimus and iliocostalis) muscles, and the CSA of the vertebral body. The relative CSA (rCSA) will be defined as the ratio between muscle CSA and vertebral body CSA and calculated for each level and side. The CSA of lean muscle in the region of interest will be defined as LeanCSA and measured on each side. The ratio of LeanCSA to the paraspinal muscle CSA will be defined as functional CSA (LeanCSA/CSA), represented as % muscle CSA and calculated for each level and side. Overall CSA, rCSA and LeanCSA will be computed as average CSA (aCSA), average rCSA (arCSA) and average LeanCSA (aLeanCSA) across all levels considering the muscle as a single unit for each side [32]. In case a different validated (semi-) automated method for assessing muscle degeneration will become available during the study duration, such method will be applied. *Stenosis severity* will be classified according to Schizas [33]. *Segmental instability* will be determined as a relative shift in anteroposterior position of two adjacent segments between the upright standing radiograph and the lying MRI of more than 3mm [34].

VII. Quality of life (EQ-5D-5L)

Overall health can be assessed with the EQ-5D-5L [35]. The EQ-5D-5L is a generic health-related instrument frequently used in clinical environments to measure quality of life. It comprises 5 questions/dimensions (mobility, self-care, usual activities, pain, depression) with Likert-scale response and a visual analogue scale where patients are asked to rate their overall health from 0 (the worst health imaginable) to 100 (the best health imaginable). Each dimension allows to choose five levels labelled as (1) 'not /no problems', (2) 'slight problems', (3) 'moderate problems', (4) 'severe problems', and (5) 'unable to' (mobility, self-care, usual activities), 'extreme' (pain/depression), or 'extremely' (anxiety/depression). Using the EQ-5D-5L, a total of 3125 different health states can be described. The final EQ index is a number between 0 and 1, with 0 indicating the poorest possible health (a state as bad as being dead) and 1 indicating the best possible health.

VIII. Swiss spinal stenosis questionnaire

The Swiss Spinal Stenosis Questionnaire is a self-report outcome questionnaire for patients with LSS that is often used in clinical studies to assess treatment outcomes in this disease. The Swiss Spinal Stenosis Questionnaire assesses the intensity of symptoms, physical function, and patient satisfaction following treatment [36].

IX. Tampa scale of kinesiophobia

The Tampa scale is a 17-item self-report questionnaire based on the assessment of fear of exercise, fear of physical activity, and fear avoidance. It consists of two subscales. While activity avoidance revolves around the reflection of activities that may increase pain or cause injury somatic focus investigates the reflection of beliefs and underlying serious conditions [37, 38].

X. Core Outcome Measures Index for the back (COMI back)

The COMI is a validated 11-item self-report questionnaire. It is used routinely worldwide for assessing the main outcomes of importance to patients back problems. However, it might be expected to be less responsive than a disease specific questionnaire [39].

XI. Upper body motion (inertial sensor data)

During all tasks, inertial sensor data will be collected. Inertial sensors (Blue Trident IMU, Vicon, Oxford, UK) will be placed on the subjects' femora, thoracic and lumbar spine and on the sternum. Accelerations and angular velocity will be captured for all movement assessments.

XII. Manuel testing for muscle strength

Selected muscles of the legs will be assessed according to Janda's M5/5 levels of strength [40]. In a sitting position: Hip flexion / M. iliopsoas (L2), Knee extension/ M. quadriceps femoris (L3). In a supine lying position: Foot extension (lift up) / M. tibialis anterior (L4), Lifting the big toe/ M extensor hallucis longus (L5). Foot extension (push down)/ M. gastrocnemius (S1).

The test for Trendelenburg signs is done by having the patient standing on one leg. If the upper body bends spontaneously to the contralateral side the test is positive and states a weakness for the m. gluteus medius, which is primarily innervated by L5 nerve root.

XIII. Physical activity level

Physical activity (PA) level will be assessed using an activity monitor (GENEActiv, Activinsights, Kimbolton, UK) during three periods of time (9 days each). The first assessment period will take place over 9 days after the baseline measurement on test day 1. The second period will take place 6 months after the spine surgery. The third period will take place for 9 days after the follow-up study visit on test day 2. The GeneActiv activity monitor is widely used in scientific research and has been shown to be highly reliable and valid [41]. The GeneActiv will be attached to the non-dominant wrist.

XIV. Back performance scale

The Back performance scale is an assessment consisting of a series of five movement activities that require mobility of the trunk. It is routinely used by physiotherapists to assess patients with back problems. The activities (sock test, pick-up test, roll-up test, fingertip-to-floor test, and lift test) all require sagittal-plane mobility and are scored from 0 (can easily do) to 3 (hard/limited to do. The test shows a good intertester agreement and test-retest reliability [42].

XV. Surgical information

Information on the surgical procedure will be extracted from the patient files:

- Scheduled decompression surgery
- Scheduled fusion of vertebrae
- Number of decompressed levels
- Number of instrumented levels
- In case of instrumentation:
    - Fusion with Cage
    - Fusion without Cage

XVI. ICHOM

The study cohorts will be described according to the guidelines by the International Consortium for Health Outcomes Measurements (ICHOM: www.ichom.org). The following information will be recorded for all participants:

- Age
- Sex
- Body mass
- Body height
- Body mass index (BMI)
- Pain medication
- Previous treatment (physiotherapy, infiltration, other)
- Own physical exercise program
- Duration of symptoms

3.4 Project plan

The project is funded by the Swiss National Foundation for a total duration of four years.

Task	2022				2023				2024				2025				2026	
	Q1	Q2	Q3	Q4	Q1	Q2	Q3	Q4	Q1	Q2	Q3	Q4	Q1	Q2	Q3	Q4	Q1	Q2
Project Initiation		X																
Obtaining ethics approval for in vivo experimental studies		X																
Baseline in vivo experimental study			X	X	X	X	X	X	X	X	X	X						
Follow-up in vivo experimental study								X	X	X	X	X	X	X	X	X	X	
Statistical analysis						X	X	X	X	X	X	X	X	X	X	X	X	X
Output – manuscripts			X			X	X	X	X	X	X	X	X	X	X	X	X	X
Recommendations clinical practice																X	X	X

3.5 Withdrawal and discontinuation

Patients have the right to withdraw from the trial at any time for any reason and without indicating a reason. The project leader also has the right to exclude patients from the trial in the event of significant adverse events, relevant protocol deviations, administrative or other reasons. In case of withdrawal, a final assessment of the information available at withdrawal will be made. The reason for withdrawal or exclusion from the trial will be recorded. A participant needs to discontinue the project in case of withdrawal of informed consent or non-compliance. Data collected before withdrawal or discontinuation will be included in subsequent analyses to account for potential data bias.

4 STATISTICS AND METHODOLOGY

4.1 Sample size calculation

To determine if PROMs correlate with dynamic compensation considering categorical covariates with 3 levels of the covariate a sample size of 111 (37 within each level) is required to detect a correlation of 0.3 at 80% power and 5% significance level. Considering a 10% drop-out rate, 122 subjects will be enrolled in the study.

4.2. Statistical analysis plan

Baseline variables will be reported using mean and standard deviation, or median and interquartile range for continuous variables, and as counts and percentages for the categorical variables. Correlation analysis will be used for analysing the association between PROMs and continuous variables, including dynamic compensation, spinal imbalance, segmental instability, muscle degeneration and fatigue, and biomechanical parameters. For measuring the association between categorical variables (e.g., the severity of stenosis) and PROMs, we will perform an analysis of variance (ANOVA). Pre- to postoperative differences in PROMs, dynamic compensation, the severity of stenosis, spinal alignment, segmental instability, muscle degeneration, muscle fatigue, and biomechanical parameters will be assessed by comparing means using paired t-tests for continuous variables, and by comparing frequencies using the chi-square test for the categorical variables. Associations of pre- to postoperative changes in PROMs with changes in static and dynamic spinal imbalance, muscle fatigue, and extent of forwarding trunk bending and lumbopelvic range of motion during walking from before to after spinal stenosis operation will be assessed by computing the difference for each variable from before and after surgery for each patient and evaluating the correlation between these computed differences. The statistical methods described here assume the normal distribution of all parameters. In case of violation of this assumption in the data we will use more appropriate statistical methods (e.g., nonparametric methods). Our predictive model building approach will be based on the seven steps of Steyerberg's [43] to construct a predictive model to predict the surgical outcome from preoperative clinical, functional, radiological, and biomechanical data and musculoskeletal model prediction of postoperative local spinal loads. Data will be checked for accuracy, inconsistencies, and plausibility of the recorded information in the data, and if required re-coding/re-grouping of certain variables will be done. We will restrict categorizing the continuous variables in the model development stage as much as possible to avoid loss of information and assess the predictive ability of the clinically relevant variables (e.g., from preoperative – spinal alignment, segmental instability, muscle degeneration, dynamic compensation, muscle fatigue, and biomechanical parameters) and variables with good/high predictive performance will be used to construct the predictive models. A different set of predictive models will be set up using the candidate predictors with the outcome PROMs. We will compare the predictive performance of the models to select the best-performed model using appropriate statistical methods (e.g., R2). The predictive models will be based on collected experimental data and also simulated data. As a few of the highly clinically relevant variables cannot be measured directly we will use the experimental data to simulate these variables. For the internal validation of the selected predictive model, we will use cross-validation.

4.3. Handling of missing data

All data will be checked for quality and completeness at the time of testing. Hence, we do not expect missing data due to technical difficulties. A 10% drop-out rate was considered in the sample size calculation (section 4.1), however, in case of an unexpected missingness after assessment of the reason for missingness in the variables we will consider using multiple imputation techniques to impute the missing information.

5 REGULATORY ASPECTS AND SAFETY

5.1 Local regulations / Declaration of Helsinki

This research project will be conducted in accordance with the protocol, the Declaration of Helsinki [3], the principles of Good Clinical Practice, the Human Research Act (HRA) and the Human Research Ordinance (HRO) [1] as well as other locally relevant regulations.

5.2 Notification of safety and protective measures (HRA Art. 15, HRO Art. 20)

If, during the research project, circumstances arise which could jeopardise the safety or health of the participants or lead to a disproportionate relationship between the risks and burdens and the benefits, all the measures required to ensure protection are to be taken without delay.
The project leader is promptly notified (within 24 hours) if immediate safety and protective measures have to be taken during the conduct of the research project. The Ethics Committee will be notified via BASEC of these measures and of the circumstances necessitating them within 7 days.

5.3 Serious events (HRO Art. 21)

If a serious event occurs, the research project will be interrupted, and the Ethics Committee notified on the circumstances via BASEC within 7 days according to HRO Art. 21[1].

5.4 Procedure for investigations involving radiation sources

EOS data will be used in this study to measure global spinal imbalance. EOS is a low-dose x-ray application used in clinical practice. The estimated (conservative estimate) effective dose will be ***0.3 mSv.*** A standard x-ray of the chest, which is considered to have minimal radiation exposure, has an effective dose of 0.1 mSv and is comparable to exposure to 10 days of natural background x-ray volume.

5.5 Amendments

Substantial changes to the project set-up, the protocol and relevant project documents will be submitted to the Ethics Committee for approval according to HRO Art. 18 before implementation. Exceptions are measures that have to be taken immediately to protect the participants.

5.6 End of project

Upon project completion or discontinuation, the Ethics Committee is notified within 90 days.

5.7 Insurance

In the event of project-related damage or injuries, the Sponsor will be liable, except for damages that are only slight and temporary; and for which the extent of the damage is no greater than would be expected in the current state of scientific knowledge (Art. 12 HRO).

6 FURTHER ASPECTS

6.1 Overall ethical considerations

Study participation is voluntary. Assessing the proposed study parameters in addition to standard clinical measures will provide additional insight into the functional limitations and the clinical course of the stenosis in our patients. This study represents a considerable time effort by the participants. However, results of the study assessments will be considered in the ongoing treatment planning and this time effort may be outweighed by the benefits not only for future patients but also for the study participants.

---

[1] A serious event is defined as any adverse event where it cannot be excluded, that the event is attributable to the sampling of biological material or the collection of health-related personal data, and which:
a. requires inpatient treatment not envisaged in the protocol or extends a current hospital stay;
b. results in permanent or significant incapacity or disability; or
c. is life-threatening or results in death.

6.2 Risk-Benefit Assessment

This project is a risk category A project. Collecting health-related personal data in this study entails minimal risks and burdens for the patient. Double sided tape will be used to attach inertial sensors and skin markers on specific anatomic landmarks. The tape may cause some skin irritation similar to a standard band-aid. Participants may experience some skin irritation at the location of skin electrodes because these locations have to be shaved and cleaned prior to electrode application. The modified Biering-Sørensen test is comparable to situations during daily, occupational or recreational activities. Overground gait analysis bears minimal risks for the patient. The MRI risk of the spine can be neglected. This standardized examination is performed in daily clinical practice. No contrast media will be given. Patients with pacemakers, neurostimulators or other incompatible devices will be excluded. All female patients prior to menopause will complete a pregnancy test before the MRI scan. If the test is positive, the patient will be excluded. EOS radiological imaging is routinely used in clinical practice. This measurement involves a minimal radiation risk (see 5.4). This study provides no direct benefit to the patient or healthy participant. Participants will be compensated with 100 CHF for each visit. Treatment of patients with sLSS is not part of the study and will not be influenced by study participation.

7 QUALITY CONTROL AND DATA PROTECTION

7.1 Quality measures

For quality assurance the Ethics Committee may visit the research sites. Direct access to the source data and all project related files and documents must be granted on such occasions.

7.2 Data recording and source data

Except for the CRFs, all data are recorded digitally. All data will be entered into a master data file created in Redcap®. Both raw and coded electronic data including EOS, MRI images and movement data will be stored and secured on a server protected by the hospital's ICT security network. All paper documentation (consent forms, questionnaires, CRFs) will be stored in a locked cabinet.

7.3 Confidentiality and coding

Project data will be handled with uttermost discretion and is only accessible to authorized personnel who require the data to fulfil their duties within the scope of the research project. On the CRFs and other project specific documents, participants are only identified by a unique participant number.

Health-related personal data will be coded using a randomly selected code from a predefined list of 6-digit codes containing letters and numbers. The code will only be broken if it is necessary to avert and immediate risk to the health of the person concerned or to guarantee the rights of the person. Data generation, transmission, storage and analysis of health-related personal data within this project will strictly follow the current Swiss legal requirements for data protection and will be performed according to the Human Research Ordinance HRO Art. 5. Health-related personal data captured during this project from participants will not be disclosed to third parties. Some of the coded motion capture data will be analyzed at ETH Zurich. Coded data originating from the study will be uploaded to a public repository according to the principles of Open Data after the termination of the project, provided that consent is explicitly given by the participant on a separate form during the informed consent procedure.

Direct access to source documents will be permitted for purposes of monitoring, audits or inspections. Project data will only be accessible to the project leader and designated team members. Any publications resulting from this project will only contain coded data and establishing a direct link between health-related data and the participants will be precluded.

7.4 Retention and destruction of study data and biological material

The data obtained in this study will be entered into an existing database on biomechanical data. These data may be used as reference data for future studies on upper extremity movement. The data will be stored indefinitely electronically on a password-protected hard drive. As specified above, the data will be coded, and the identification key will be locked and only accessible to the project leader and designated team members. Data of participants who used their right to withdraw their consent for future data use will be destroyed upon study completion.

8. FUNDING / PUBLICATION / DECLARATION OF INTEREST

This study is mainly funded by the Swiss National Science Foundation (SNSF #204461). The SNSF is not involved in any aspect of the project.

Local progress reports will be used to disseminate information obtained in this study to healthcare professionals at our institution. The results of this study will be published at scientific conferences and in peer-reviewed international scientific journals. Any publications resulting from this project will only contain coded data and establishing a direct link between health related data and the participants will be precluded.

Interested third parties may contact the project leader. Data will not be shared with third parties with lower data protection standards than CH or EU. Only coded data but not the key to the code may be shared.

We declare no conflict of interest.

9. REFERENCES

1. Ignasiak, D., A. Rueger, and S.J. Ferguson, *Multi-segmental thoracic spine kinematics measured dynamically in the young and elderly during flexion.* Hum Mov Sci, 2017. **54**: p. 230-239.
2. Völlner, F. and J. Grifka, *Biomechanische Aspekte der präoperativen Planung: Was ist wirklich wichtig?* Der Orthopäde, 2019. **48**(1): p. 44-49.
3. *Ordinance on Human Research with the Exception of Clinical trials (HRO)* Available from: https://www.admin.ch/opc/en/classified-compilation/20121177/index.html
4. *Human Research Act (HRA)*. Available from: www.admin.ch/opc/en/classified-compilation/20121176/201401010000/810.305.pdf.
5. *Declaration of Helsinki* Available from: https://www.wma.net/policies-post/wma-declaration-of-helsinki-ethical-principles-for-medical-research-involving-human-subjects.
6. Rampersaud, Y.R., et al., *Assessment of health-related quality of life after surgical treatment of focal symptomatic spinal stenosis compared with osteoarthritis of the hip or knee.* Spine J, 2008. **8**(2): p. 296-304.
7. Ciol, M.A., et al., *An assessment of surgery for spinal stenosis: time trends, geographic variations, complications, and reoperations.* J Am Geriatr Soc, 1996. **44**(3): p. 285-90.
8. Deyo, R.A., et al., *United States trends in lumbar fusion surgery for degenerative conditions.* Spine (Phila Pa 1976), 2005. **30**(12): p. 1441-5; discussion 1446-7.
9. Fanuele, J.C., et al., *The impact of spinal problems on the health status of patients: have we underestimated the effect?* Spine (Phila Pa 1976), 2000. **25**(12): p. 1509-14.
10. Jespersen, A.B. and M. Gustafsson, *Correlation between the Oswestry Disability Index and objective measurements of walking capacity and performance in patients with lumbar spinal stenosis: a systematic literature review.* Eur Spine J, 2018. **27**(7): p. 1604-1613.
11. Fortin, M., et al., *Association between paraspinal muscle morphology, clinical symptoms and functional status in patients with lumbar spinal stenosis.* Eur Spine J, 2017. **26**(10): p. 2543-2551.
12. Norden, J., et al., *Objective measurement of free-living physical activity (performance) in lumbar spinal stenosis: are physical activity guidelines being met?* Spine J, 2017. **17**(1): p. 26-33.
13. Byrnes, S.K., et al., *Änderung des Gangbildes nach Dekompressionsoperation bei Patienten mit lumbaler Spinalkanalstenose: Ein-Jahres-Follow-Up mit Gang- und Attraktoranalyse basierend auf Inertialsensoren [Change in gait pattern after decompression surgery in patients with lumbar spinal stenosis: 1-year follow-up with gait and attractor analysis based on inertial sensors]*, in *2nd GAMMA Congress*. 2018: Hamburg, Germany.
14. Loske, S., et al., *Decompression surgery improves gait quality in patients with symptomatic lumbar spinal stenosis*, in *105th Congress of the Swiss Society of Surgeons (SGC)*. 2018: Basel, Switzerland.
15. Bumann, H., et al., *Severity of degenerative lumbar spinal stenosis affects pelvic rigidity during walking.* Spine J, 2019.
16. Thornes, E., H.S. Robinson, and N.K. Vollestad, *Dynamic balance in patients with degenerative lumbar spinal stenosis; a cross-sectional study.* BMC Musculoskelet Disord, 2018. **19**(1): p. 192.
17. Farrokhi, M.R., et al., *Spinal sagittal balance and spinopelvic parameters in patients with degenerative lumbar spinal stenosis; a comparative study.* Clin Neurol Neurosurg, 2016. **151**: p. 136-141.

18. Zingg, P.O. and N. Boos, *Lumbar spinal stenosis*, in *Spinal Disorders Fundamentals of Diagnosis and Treatment*, N. Boos and M. Aebi, Editors. 2008, Springer-Verlag Berlin Heidelberg.
19. Dreischarf, M., et al., *Estimation of loads on human lumbar spine: A review of in vivo and computational model studies.* J Biomech, 2016. **49**(6): p. 833-845.
20. Ignasiak, D., S.J. Ferguson, and N. Arjmand, *A rigid thorax assumption affects model loading predictions at the upper but not lower lumbar levels.* J Biomech, 2016. **49**(13): p. 3074-3078.
21. Ignasiak, D., et al., *Thoracolumbar spine loading associated with kinematics of the young and the elderly during activities of daily living.* J Biomech, 2018. **70**: p. 175-184.
22. Harris, P.A., et al., *Research electronic data capture (REDCap)—A metadata-driven methodology and workflow process for providing translational research informatics support.* Journal of Biomedical Informatics, 2009. **42**(2): p. 377-381.
23. Biering-Sørensen, F., *Physical measurements as risk indicators for low-back trouble over a one-year period.* Spine, 1984. **9**(2): p. 106-119.
24. Leboeuf, F. *CGM 2.3 Removal of thigh wands*. 2018 [cited 2022 25.02.2022]; Available from: https://pycgm2.github.io/pages/CGM23-Overview.html.
25. Mannion, A.F., et al., *Development of a German version of the Oswestry Disability Index. Part 2: sensitivity to change after spinal surgery.* Eur Spine J, 2006. **15**(1): p. 66-73.
26. Mannion, A.F., et al., *Development of a German version of the Oswestry Disability Index. Part 1: cross-cultural adaptation, reliability, and validity.* European Spine Journal, 2006. **15**(1): p. 55-65.
27. Ignasiak, D., S. Dendorfer, and S.J. Ferguson, *Thoracolumbar spine model with articulated ribcage for the prediction of dynamic spinal loading.* J Biomech, 2016. **49**(6): p. 959-966.
28. Duval-Beaupere, G., C. Schmidt, and P. Cosson, *A Barycentremetric study of the sagittal shape of spine and pelvis: the conditions required for an economic standing position.* Ann Biomed Eng, 1992. **20**(4): p. 451-62.
29. Boutellier, A., et al., *Trunk muscle function and its association with functional limitations in sedentary occupation workers with and without chronic nonspecific low back pain.* Journal of Back and Musculoskeletal Rehabilitation, 2021. **Preprint**: p. 1-9.
30. Fortin, M., et al., *Evaluation of an automated thresholding algorithm for the quantification of paraspinal muscle composition from MRI images.* Biomed Eng Online, 2017. **16**(1): p. 61.
31. Fortin, M. and M.C. Battie, *Quantitative paraspinal muscle measurements: inter-software reliability and agreement using OsiriX and ImageJ.* Phys Ther, 2012. **92**(6): p. 853-64.
32. Mandelli, F., et al., *Assessing fatty infiltration of paraspinal muscles in patients with lumbar spinal stenosis: Goutallier classification and quantitative MRI measurements.* Frontiers in Neurology, in review.
33. Schizas, C., et al., *Qualitative grading of severity of lumbar spinal stenosis based on the morphology of the dural sac on magnetic resonance images.* Spine (Phila Pa 1976), 2010. **35**(21): p. 1919-24.
34. Jang, S.Y., et al., *Radiographic parameters of segmental instability in lumbar spine using kinetic MRI.* J Korean Neurosurg Soc, 2009. **45**(1): p. 24-31.
35. Herdman, M., et al., *Development and preliminary testing of the new five-level version of EQ-5D (EQ-5D-5L).* Qual Life Res, 2011. **20**(10): p. 1727-36.

36. Wertli, M.M., et al., *Cross-cultural adaptation of the German version of the spinal stenosis measure.* European spine journal : official publication of the European Spine Society, the European Spinal Deformity Society, and the European Section of the Cervical Spine Research Society, 2014. **23**.
37. Gregg, C.D., et al., *The relationship between the Tampa Scale of Kinesiophobia and low back pain rehabilitation outcomes.* Spine J, 2015. **15**(12): p. 2466-71.
38. Hapidou, E.G., et al., *Fear and Avoidance of Movement in People with Chronic Pain: Psychometric Properties of the 11-Item Tampa Scale for Kinesiophobia (TSK-11).* Physiother Can, 2012. **64**(3): p. 235-41.
39. Impellizzeri, F.M., et al., *The use of the Core Outcome Measures Index (COMI) in patients undergoing total knee replacement.* Knee, 2017. **24**(2): p. 372-379.
40. Janda, V., *Manuelle Muskelfunktionsdiagnostik*. Vol. 4. 2009: Urban & Fischer.
41. Pavey, T.G., et al., *The validity of the GENEActiv wrist-worn accelerometer for measuring adult sedentary time in free living.* J Sci Med Sport, 2016. **19**(5): p. 395-9.
42. Magnussen, L., L.I. Strand, and H. Lygren, *Reliability and validity of the back performance scale: observing activity limitation in patients with back pain.* Spine (Phila Pa 1976), 2004. **29**(8): p. 903-7.
43. Steyerberg, E.W., *Clinical prediction models*. 2019, Cham: Springer International Publishing.
