# Supplementary material for: The role of muscle degeneration and spinal balance in the pathophysiology of lumbar spinal stenosis: Study protocol of a translational approach combining in vivo biomechanical experiments with clinical and radiological parameters
Source: PLoS One. 2023 Oct 27;18(10):e0293435. doi: 10.1371/journal.pone.0293435 (PMC10610482; doi:10.1371/journal.pone.0293435)
Supplement: S5 File — (PDF) [file pone.0293435.s006.pdf]

[redacted]

EKNZ

Ethikkommission
Nordwest- und
Zentralschweiz

Präsident
Prof. Christoph Beglinger
Vizepräsidenten
Dr. Angela Frotzler
Dr. Marco Schärer

PD Dr Cordula Netzer
University Hospital Basel
Spitalstrasse 21
4031 Basel

Basel, 28. März 2023 / LF

Verfügung der Ethikkommission Nordwest- und Zentralschweiz (EKNZ)

**Wesentliche Änderung**	**Amendment 01 vom 27. März 2023 (Protokoll Version 3)**
**eingereicht am**	27.03.2023
**Project-ID**	2022-01170
**Projekttitel**	RoLSSroice - Role of spinal load in the pathophysiology of lumbar spinal stenosis: A translational approach combining clinical and radiological parameters, in vivo biomechanical experiments and advanced in silico musculoskeletal modeling
**Master-/Doktorarbeit von**	Koch, David
**Projektleitung**	PD Dr Cordula Netzer
**Sponsor**	USB, Prof Stefan Schären
**Zentren**	PD Dr Cordula Netzer, University Hospital Basel, Basel

Entscheidverfahren

☐ vereinfachtes Verfahren ☒ Präsidialverfahren

Entscheid

PD Dr Cordula Netzer, University Hospital Basel, Basel

☒ Die Bewilligung wird erteilt
☐ Die Bewilligung wird mit Auflagen erteilt
☐ Die Bewilligung kann noch nicht erteilt werden
☐ Die Bewilligung wird nicht erteilt

Gebühren

Betrag **CHF 250.--** **Tarifcode** **3.3.1**
Gemäss der geltenden Gebührenordnung von swissethics.

Rechtsmittelbelehrung

Gegen diesen Entscheid kann an den Regierungsrat des Kantons Basel-Stadt (Rathaus, Marktplatz 9, 4051 Basel) rekurriert werden. Der Rekurs ist innert 10 Tagen seit Eröffnung des Entscheides bei der Rekursinstanz anzumelden; innert 30 Tagen, vom gleichen Zeitpunkt an gerechnet, ist die Rekursbegründung einzureichen, welche die Anträge und deren Begründung mit Angabe der Beweismittel zu enthalten hat. Bei völliger oder teilweiser Abweisung des Rekurses können die Kosten der Rekurrentin

respektive dem Rekurrenten ganz oder teilweise auferlegt werden.

Kopie an

☐	BAG	
☒	Sponsor	USB, Stefan.Schaeren@usb.ch
☒	Andere	Ilona Ahlborn, ilona.ahlborn@usb.ch<br>David Koch, david.koch@usb.ch

Die Ethikkommission bestätigt, dass sie nach ICH-GCP arbeitet.

Unterschrift

i.V. [redacted]

Prof. Dr. med. Christoph Beglinger
Präsident

Anhang:
1. Bedeutung der möglichen Entscheide
2. Liste der Dokumente, eingereicht am 27.03.2023

Anhang 1

Bedeutung der möglichen Entscheide

Die Bewilligung wird erteilt: Das Vorhaben kann gemäss der oben aufgeführten wesentlichen Änderung weitergeführt werden.

Die Bewilligung wird mit Auflagen erteilt: Das Forschungsprojekt kann gemäss der oben aufgeführten wesentlichen Änderung durchgeführt werden. Die Auflagen sind innert 30 Tagen zu erfüllen.

Die Bewilligung kann noch nicht erteilt werden: Das Vorhaben kann noch nicht gemäss der oben aufgeführten wesentlichen Änderung durchgeführt werden. Die Bedingungen sind zu erfüllen. Die revidierte Änderung wird nach Einreichung von der Ethikkommission geprüft.

Die Bewilligung wird nicht erteilt: Das Vorhaben kann in der vorliegenden Form nicht durchgeführt werden. Eine Neueinreichung der wesentlichen Änderung ist möglich.

Anhang 2

Liste der Dokumente, eingereicht am 27.03.2023

PD Dr Cordula Netzer, University Hospital Basel, Basel

Dokument	Kategorie	Dok.Datum	Version
rolssroice-cover-letter-swiss-ethics-v3-clean.pdf	1. Cover Letter	27/03/2023	
rolssroice-studieninformation-patient-v3-tc.docx	3. ICF	27/03/2023	3
rolssroice-studieninformation-patient-v3-clean.docx	3. ICF	27/03/2023	3
rolssroice-signatureform-v3-signed.pdf	4. Study plan	27/03/2023	1
rolssroice-studprot-v3-tc.docx	4. Study plan	27/03/2023	3
rolssroice-studprot-v3-clean.docx	4. Study plan	27/03/2023	3
rolssroice-crf-v2-clean.docx	5. CRF	27/03/2023	2
rolssroice-crf-v2-tc.docx	5. CRF	27/03/2023	2
