# Supplementary material for: The role of muscle degeneration and spinal balance in the pathophysiology of lumbar spinal stenosis: Study protocol of a translational approach combining in vivo biomechanical experiments with clinical and radiological parameters
Source: PLoS One. 2023 Oct 27;18(10):e0293435. doi: 10.1371/journal.pone.0293435 (PMC10610482; doi:10.1371/journal.pone.0293435)
Supplement: S6 File — (PDF) [file pone.0293435.s007.pdf]

EKNZ

Ethikkommission
Nordwest- und
Zentralschweiz

Präsident
Prof. Christoph Beglinger
Vizepräsidenten
Dr. Angela Frotzler
Dr. Marco Schärer

To whom it may concern

Basel, August 15th, 2022 / SK

Confirmation of ethic approval for the project "RoLSSroice - Role of spinal load in the pathophysiology of lumbar spinal stenosis: A translational approach combining clinical and radiological parameters, in vivo biomechanical experiments and advanced in silico musculoskeletal modeling" - BASEC ID 2022-01170.

Dear Sir, dear Madam,

We hereby confirm that the project "RoLSSroice - Role of spinal load in the pathophysiology of lumbar spinal stenosis: A translational approach combining clinical and radiological parameters, in vivo biomechanical experiments and advanced in silico musculoskeletal modeling", initiated by PD Dr. Cordula Netzer, has been approved by the Ethics Committee Northwest and Central Switzerland (EKNZ) on August 09th, 2022.

Yours sincerely,

i.V.i. [redacted]

Prof. Dr. Christoph Beglinger, MD
President of the Ethics Committee
Northwest and Central Switzerland / EKNZ
